# Effectiveness of Long-Acting Injectable Antipsychotics Versus Oral Antipsychotics in People With Bipolar Disorder: A Systematic Review and Meta-Analysis of Observational Studies: Efficacité des antipsychotiques injectables à action prolongée par rapport aux antipsychotiques oraux chez les personnes atteintes de troubles bipolaires : revue systématique et méta-analyse d'études observationnelles

**DOI:** 10.1177/07067437251412576

**Published:** 2026-01-30

**Authors:** Elias Wagner, Saguna Katyal, In Ok Lee, Sabah Tasnim, Hajar El Wadia, Matin Mortazavi, Juan Antonio García-Carmona, Alkomiet Hasan, Ian Colman, Heidi Taipale, Jari Tiihonen, Christoph U Correll, Mikkel Højlund, Marco Solmi

**Affiliations:** 1 Evidence-Based Psychiatry and Psychotherapy, Faculty of Medicine, 39694 University of Augsburg, Augsburg, Germany; 2 Department of Psychiatry, Psychotherapy and Psychosomatics, Medical Faculty, University of Augsburg, Augsburg, Germany; 3 School of Epidemiology and Public Health, 6363 University of Ottawa, Ottawa, Canada; 4 Department of Neurology, 525168 Santa Lucía University Hospital, Murcia, Spain; 5 Group of Clinical and Experimental Pharmacology, Institute for Biomedical Research of Murcia (IMIB), Murcia, Spain; 6 Faculty of Pharmacy and Nutrition, San Antonio Catholic University of Murcia (UCAM), Murcia, Spain; 7 Department of Psychiatry, Psychotherapy and Psychosomatics Augsburg, DZPG (German Center for Mental Health), Augsburg, Germany; 8 Department of Clinical Neuroscience, 27106 Karolinska Institutet, Stockholm, Sweden; 9 Center for Psychiatry Research, Stockholm City Council, Stockholm, Sweden; 10 Department of Forensic Psychiatry, University of Eastern Finland, Niuvanniemi Hospital, Kuopio, Finland; 11 School of Pharmacy, University of Eastern Finland, Kuopio, Finland; 12 Department of Psychiatry, 14903 Zucker Hillside Hospital, Glen Oaks, NY, USA; 13 Departments of Psychiatry and Molecular Medicine, Donald and Barbara Zucker School of Medicine at Hofstra/Northwell, Hempstead, New York, USA; 14 Department of Child and Adolescent Psychiatry, Charité Universitätsmedizin Berlin, Berlin, Germany; 15 German Center for Mental Health (DZPG), Partner Site Berlin, Berlin, Germany; 16 Department of Psychiatry Aabenraa, 91944 Mental Health Services Region of Southern Denmark, Aabenraa, Denmark; 17 Clinical Pharmacology, Pharmacy, and Environmental Medicine, Department of Public Health, University of Southern Denmark, Odense, Denmark; 18 SCIENCES Lab, Department of Psychiatry, 6363 University of Ottawa, Ontario, Canada; 19 Regional Centre for the Treatment of Eating Disorders and On Track: The Champlain First Episode Psychosis Program, Department of Mental Health, The Ottawa Hospital, Ontario, Canada; 20 Ottawa Hospital Research Institute (OHRI), Ottawa, Ontario, Canada

**Keywords:** bipolar disorder, long-acting injectable, cohort study, mirror-image study, hospitalisation

## Abstract

**Background:**

Randomised trials suggest long-acting injectable antipsychotics (LAIs) may outperform oral antipsychotics (OAPs) regarding adherence and relapse prevention in bipolar disorder (BD). We aimed to compare the effectiveness and tolerability of LAIs versus OAPs in observational studies.

**Methods:**

Searching MEDLINE/Embase/PsycINFO until March-25-2025, we conducted a systematic review and random-effects meta-analysis (pre-registered protocol: https://osf.io/gkwrp) of observational studies comparing LAIs versus OAPs in people with BD (primary outcome = study-defined relapse/psychiatric hospitalisation).

**Results:**

Seventeen studies (4 = cohort, 13 = mirror-image studies; 6186/3676 participants with BD, respectively, high-quality per Newcastle–Ottawa Scale Score ≥7 = 47.1%) were included. The relative risk (RR) for study-defined relapse/psychiatric hospitalisation was significantly lower with LAIs versus OAPs in cohort (*k* = 4, RR = 0.63, 95% confidence interval (CI) = 0.44;0.90, *P* = 0.026) and mirror-image studies (*k* = 5, RR = 0.46, 95% CI = 0.28;0.77, *P* = 0.013). LAIs were not significantly superior to OAPs in high-quality cohort studies (*k* = 3, *P* = 0.78) but in those adjusted for >5 factors (*k* = 2, RR = 0.56, 95% CI = 0.37;0.84, *P* = 0.006) nor in high-quality mirror-image studies (*k* = 2, *P* = 0.38), but in each second-generation antipsychotic-LAIs study (aripiprazole-LAI: *k* = 2, risperidone-LAI: *k* = 1) (*k* = 3, RR = 0.40, 95% CI = 0.20;0.80, *P* = 0.03). In cohort studies, LAIs and OAPs did not differ regarding psychiatric hospitalisations (*k* = 3, *P* = 0.078) but data on discontinuation and mortality risk were lacking/not meta-analysable. In mirror-image studies, LAIs were associated with significantly lower psychiatric (*k* = 4, RR = 0.50, 95% CI = 0.25;0.99, *P* = 0.048) and depression-related (*k* = 2, RR = 0.46, 95% CI = 0.24;0.86, *P* = 0.014), but not mania-related hospitalisation risk (*k* = 2, *P* = 0.075). LAIs were associated with fewer psychiatric hospitalisations (*k* = 7, SMD = −1.73, 95% CI = −2.88;−0.57, *P* = 0.011), hospitalisation days (*k* = 9, SMD = −1.35, 95% CI = −2.19;−0.52, *P* = 0.006), mania-related hospitalisations (*k* = 3, SMD = −0.87, 95% CI = −1.19;−0.56, *P* < 0.001) and manic episodes (*k* = 3, SMD = −1.13, 95% CI = −2.00;−0.26, *P* = 0.03), but not any mood (*k* = 4, *P* = 0.13) or depressive episodes (*k* = 3, *P* = 0.14). Tolerability outcomes were missing, and GRADE certainty-of-evidence was *“*low” to “very low”.

**Discussion:**

LAIs were superior versus OAPs in preventing relapse/hospitalisation in cohort and mirror-image studies, in the latter particularly for mania-related outcomes. More robust mirror-image and controlled cohort studies are needed to better assess the effectiveness and tolerability of LAI antipsychotics in BD.

## Plain Language Summary Title

Effectiveness of Long-Acting Injectable Antipsychotics versus Oral Antipsychotics in People with Bipolar Disorder: A Systematic Review and Meta-Analysis of Observational Studies

**Plain Language Summary:**

Clinical trials have suggested that long-acting injectable antipsychotics (LAIs) may help people with bipolar disorder stay on their medication and reduce relapse risk better than oral antipsychotics. This review looked at real-world studies to see how well LAIs work compared to oral antipsychotics in everyday clinical settings.

We searched several medical databases up to March 2025 and included 17 studies that compared LAIs and OAPs in people with bipolar disorder. These studies followed people over time or compared outcomes before and after switching from oral to injectable medication. The main outcome was relapse or psychiatric hospitalization. Across studies, people using LAIs had a lower risk of relapse or psychiatric hospitalization compared to those taking oral antipsychotics. This benefit was seen in both types of studies (cohort and mirror-image).

In studies that adjusted for more factors or focused on second-generation LAIs (like aripiprazole or risperidone), LAIs appeared particularly effective. LAIs were linked to fewer psychiatric hospitalizations overall, fewer days spent in hospital, and fewer manic episodes.

There was no clear difference for depressive episodes. Information on side effects or tolerability was often missing. The overall certainty of the evidence was rated as low to very low, meaning more high-quality studies are needed. Long-acting injectable antipsychotics may help people with bipolar disorder stay well and avoid relapse or hospitalization better than oral medications, especially for mania. However, because most existing studies have limitations, more rigorous research is needed to confirm these findings and to understand the side effects of LAIs compared to oral antipsychotics.

## Résumé

**Contexte:**

Selon des essais à répartition aléatoire, les antipsychotiques injectables à action prolongée (APIAP) pourraient donner de meilleurs résultats sur le plan de l'observance et de la prévention des rechutes que les antipsychotiques oraux (APO) pour le traitement des troubles bipolaires (TB). Nous avons cherché à comparer l'efficacité et la tolérabilité des APIAP par rapport aux APO dans des études observationnelles.

**Méthodologie:**

En faisant des recherches dans les bases de données MEDLINE/Embase/PsycINFO jusqu'au 25 mars 2025, nous avons procédé à une revue systématique et à une méta-analyse des effets aléatoires (protocole enregistré au préalable : https://osf.io/gkwrp) dans des études observationnelles comparant les APIAP et les APO chez des personnes atteintes de TB (critère d'évaluation principal : rechutes/hospitalisations psychiatriques définies dans le cadre de l'étude).

**Résultats:**

Dix-sept études (4 études de cohorte, 13 études en miroir; 6 186/3 676 participants atteints de TB respectivement, haute qualité définie en fonction d'un score ≥ 7sur l'échelle Newcastle-Ottawa = 47,1 %) ont été incluses. Le risque relatif (RR) de rechutes/hospitalisations psychiatriques définies dans le cadre de l'étude était significativement plus faible avec les APIAP qu'avec les APO dans les études de cohorte (k = 4, RR = 0,63, intervalle de confiance [IC] à 95 % = 0,44; 0,90, *p* = 0,026) et les études en miroir (k = 5, RR = 0,46, IC à 95 % = 0,28; 0,77, *p* = 0,013). Les APIAP n'étaient pas significativement supérieurs aux APO dans les études de cohorte de haute qualité (k = 3, *p* = 0,78), mais l'étaient dans les études ajustées pour plus de 5 facteurs (k = 2, RR = 0,56, IC à 95 % = 0,37 à 0,84, *p* = 0,006); ils ne l'étaient pas dans les études en miroir de haute qualité (k = 2, *p* = 0,38), mais l'étaient dans chaque étude portant sur un APIAP de deuxième génération (aripiprazole AP : k = 2, rispéridone AP : k = 1) (k = 3, RR = 0,40, IC à 95 % = 0,20 à 0,80, *p* = 0,03). Dans les études de cohorte, les APIAP et les APO ne différaient pas sur le plan des hospitalisations psychiatriques (k = 3, p = 0,078), mais les données sur les résultats étaient insuffisantes ou ne pouvaient pas faire l'objet d'une méta-analyse. Dans les études en miroir, les APIAP étaient associés à un risque beaucoup plus faible d'hospitalisation liée à un trouble psychiatrique (k = 4, RR = 0,50, IC à 95 % = 0,25 à 0,99, *p* = 0,048) et liée à une dépression (k = 2, RR = 0,46, IC à 95 % = 0,24 à 0,86, *p* = 0,014), mais pas sur le plan du risque d'hospitalisation liée à un trouble maniaque (k = 2, *p* = 0,075). Les APIAP étaient associés à une diminution des hospitalisations liées à un trouble psychiatrique (k = 7, DMS = -1,73, IC à 95 % = -2,88 à -0,57, *p* = 0,011), des jours d'hospitalisation (k = 9, DMS = -1,35, IC à 95 % = -2,19 à -0,52, *p* = 0,006), des hospitalisations liées à un trouble maniaque (k = 3, DMS = -0,87, IC à 95 % = -1,19 à -0,56, *p* < 0,001) et des épisodes maniaques (k = 3, DMS = -1,13, IC à 95 % = -2,00 à -0,26, *p* = 0,03), mais pas des troubles de l'humeur (k = 4, *p* = 0,13) ni des épisodes dépressifs (k = 3, *p* = 0,14). Les résultats en matière de tolérabilité n'étaient pas suffisants, et le DEGRÉ de certitude des données probantes était *«* faible » à « très faible ».

**Discussion:**

Les APIAP étaient supérieurs aux APO pour ce qui est de la prévention des rechutes et des hospitalisations dans les études de cohorte et en miroir, dans ces dernières en particulier pour les résultats liés à la manie. Des études de cohortes contrôlées et en miroir plus robustes sont nécessaires pour permettre une meilleure évaluation de l'efficacité et de la tolérabilité des antipsychotiques injectables à action prolongée (APIAP) pour le traitement des TB.

## Introduction

Bipolar disorder (BD) is a relapsing disorder, often resulting in alternating manic and depressive mood episodes.^1–3^ Evidence-based pharmacological treatment comprises conventional mood stabilisers (antiepileptics or lithium), oral second-generation antipsychotics (SGAs), each in monotherapy or in various combinations.^1,4^ However, the adherence to oral treatment regimens remains low^5^ and pharmacological non-adherence has been associated with poor clinical outcomes.^6^ Two long-acting injectable antipsychotics (LAIs), risperidone-LAI and aripiprazole monohydrate-LAI one-monthly and two-monthly, have been approved by the U.S. Food and Drug Administration (FDA) for maintenance treatment of people with BD.^7–10^ Nonetheless, despite LAIs being off-label used in European clinical practice, none have been approved by the European Medicines Agency (EMA). Current literature demonstrates the efficacy of LAIs in improving medication adherence, preventing relapse, decreasing hospitalisation rates and reducing the overall burden on the healthcare system.^11^ A previous study demonstrated that people with BD on LAIs were 19% less likely to stop taking their medication compared to people taking oral antipsychotic medications (OAPs).^12^ Clinicians cite the lack of adherence to antipsychotic medication as a major reason for LAI prescription in BD.^13^ Despite the fact that LAIs are superior to placebo for relapse and all-cause discontinuation in people with BD,^11,14^ and that they are also indicated in international guidelines as first- or second-line treatment in the maintenance phase of BD, their use in clinical practice has remained low.^4^ This situation is likely at least partially due to the limited body of evidence related to LAI use among people with BD, as well as insufficient evidence from RCTs supporting superiority of LAIs over OAPs in preventing relapse in BD.^15^ However, negative findings from RCTs not showing superiority of LAIs versus OAPs or other active control conditions should be interpreted with caution, as has been discussed in schizophrenia.^16^ The representativeness gap between RCTs and naturalistic studies demonstrated clearly in schizophrenia^17,18^ calls for a thorough investigation of outcomes in real-world studies, especially since adherence to OAPs observed in RCTs may not reflect pharmacological real-world adherence, which is likely significantly lower in patients who are often sicker, not incentivised to return for visits and who are not assessed for non-adherence or given mediations at appointments. In addition, the follow-up times in RCTs are relatively short, usually a few weeks to months, which is not sufficient time to study long-term medication adherence in people with BD.^12^ Evidence from cohort studies, including data from chart reviews and administrative databases, can complement findings from RCTs to better inform patient and provider decision-making. Moreover, mirror-image studies (i.e., pre–post studies after a switch from OAPs to LAIs) have the advantage of using participants as their own control, which can reduce residual individual confounding and assess the effectiveness of LAIs in the real world. Therefore, an assessment of real-world evidence regarding the effectiveness of LAIs among people with BD is needed to inform the current treatment guidelines for the short- and long-term management of BD. A systematic review without meta-analysis reported evidence suggesting superiority of LAIs over non-LAI in preventing relapses, access to emergency rooms and hospital days.^10^ A recent meta-analysis of mirror-image studies in BD found that use of LAIs is an effective strategy to improve major clinical outcomes in people with BD.^10^ However, this meta-analysis did not include all possible outcomes reported in the original studies. Moreover, additional mirror-image studies were recently published,^19–22^ and to our knowledge, no meta-analysis of cohort studies is available. Hence, we conducted an updated meta-analysis of cohort and mirror-image studies comparing LAIs versus OAPs in people with BD, focusing on study-defined relapse as the primary outcome, but without any outcome restrictions.

## Methods

The protocol for this systematic review was registered in Open Science Framework (OSF) (https://osf.io/gkwrp). This study is reported as per PRISMA 2020 guidelines and the completed PRISMA checklist is available in the Supplementary Material.^23^

Any deviations from the original protocol are reported in the Supplementary Table S7.

### Eligibility Criteria

We included observational studies (cohort or mirror-image studies) that recruited people of any age diagnosed with BD-I or BD-II according to DSM- or ICD-any version, or clinical diagnosis, in all phases of BD (mania, bipolar depression, maintenance/euthymia), comparing LAIs versus OAPs. The OAP group only included people on OAPs without LAIs, whereas the LAI group could also include adjunctive OAPs. In studies conducting analyses within subject s, the people with BD were compared to themselves when they were versus when they were not taking any LAIs. In studies containing populations with different types of mental illnesses (e.g., schizophrenia and BD), we extracted results specific to patients with BD. Otherwise, study authors were contacted, and if no BD specific results were obtained, the study was excluded. All corresponding authors from cohort studies were contacted to provide additional data on all-cause discontinuation estimates and mortality rates. Studies with within-individual design were labelled as mirror-image studies, whereas cohort studies with between-individual design were labelled as cohort studies.

### Outcomes

The primary outcome was risk of study-defined relapse (which can include psychiatric hospitalisation) or psychiatric hospitalisation whichever was available in the original studies. Key secondary outcomes were hospitalisation risk, number of hospitalisations (due to any cause or related to mania or depression), mean hospitalisation days and mean number of (any) mood episodes. Secondary outcomes were the risk and mean number of emergency department (ED) visits, risk of symptomatic relapse and the mean time to relapse. No language or date restrictions were applied. All outcomes were rated according to GRADE^24^ regarding certainty of evidence. In GRADE, certainty in evidence from pooled outcomes was downgraded from “low” to “very low” in case of high risk of bias (e.g., selection bias), inconsistency (e.g., *I*^2^ > 50%) or imprecision (e.g., confidence intervals including both benefit and harm).

### Information Sources and Search Strategy

Electronic searches were conducted in Ovid, MEDLINE, Embase and PsycINFO from database inception to March 25, 2025. The search strategy was peer-reviewed by a librarian at The Ottawa Hospital. The search was developed in MEDLINE using medical subject headings (MeSH), such as “bipolar disorder” and “delayed release formulation” in combination with relevant key words, such as “manic depression”, “manic depressive psychosis”, “manic disorder”, “bipolar depression”, “long-acting injectable”, “depot antipsychotic”, “intramuscular injection” and more. The full search strategy is shown in the Supplementary Methods. Manual searches were also conducted in Google.

### Study Selection and Data Extraction

Seven co-authors (SK, IOL, ST, HEW, EW, MH, MM) independently conducted the title and abstract screening, whereby each study was screened in duplicate. Any studies with disagreements between authors at this stage were moved to full text review, and any remaining disagreements during this phase were resolved by a 3rd co-author (MS).

Two co-authors independently extracted data (EW, MH) and assessed study quality with the Newcastle–Ottawa Scale (NOS) for cohort studies.^25^ The NOS evaluates study quality across the three key domains: selection, comparability between groups and outcome ascertainment. A NOS score of ≥7 was the cut-off to select studies rated as “good”. Any disagreements during this phase were resolved by a 3rd co-author (MS).

### Effect Measures

For time-to-event variables, hazard ratios (HRs) and 95% confidence intervals (CIs) were collected when available. For continuous outcomes, mean and standard deviation were extracted, as well as mean differences and 95% CIs if available. For dichotomous outcomes, effect measures such as risk ratios (RRs), odds ratios (ORs) and 95% CIs were extracted when available. Otherwise, the number of participants with the respective outcome was extracted. Adjusted measures were obtained when available. Metaconvert was used to convert reported original data to standardised mean differences (SMDs), mean differences (MDs) or RRs.^26^ HRs were pooled with RRs as both are ratio measures. To assess the robustness of pooled MDs and SMDs to different assumptions about pre–post correlations, we conducted a sensitivity analysis using plausible correlation coefficients (*r* = 0.3, 0.5, 0.7) when calculating MDs and SMDs from studies reporting only group-level pre- and post-intervention means with corresponding SDs. Hartung–Knapp adjustments were performed if the number of studies was >2 and <10 and tau^2^ > 0 due to a limited number of studies in the respective analyses and overall high heterogeneity.^27^ Heterogeneity across studies was assessed using the *I*^2^ statistic, which describes the percentage of total variation due to heterogeneity rather than chance, and the tau^2^ statistic, which estimates the between-study variance.^28^

### Main and Sensitivity Analyses and Meta-Regression Analyses

Main analyses pooled results from mirror-image studies separately from cohort studies as each systematically has a different comparator to patients treated with LAIs (mirror-image study: the same person on OAP; cohort study: other person with BD and on OAPs). Sensitivity analyses were conducted on studies with a lower risk of bias (NOS rating ≥7) and studies only using second-generation antipsychotics in relation to specific study type. For meta-regression analyses, the following variables were extracted as baseline markers in cohort studies if available: illness duration, number of previous suicide attempts, number of previous mood episodes, number of previous psychiatric, somatic and all-cause admissions, proportions of people with comorbid anxiety disorders, post-traumatic stress disorder, personality disorder, psychosis and substance use disorder, as well as the proportion of concomitant mood-stabilising treatment and people with rapid cycling BD and the mean body mass index (BMI). Furthermore, information was extracted if and for how many covariates the analysis was adjusted.

## Results

### Study Selection and Description of Included Studies

**Figure 1.**
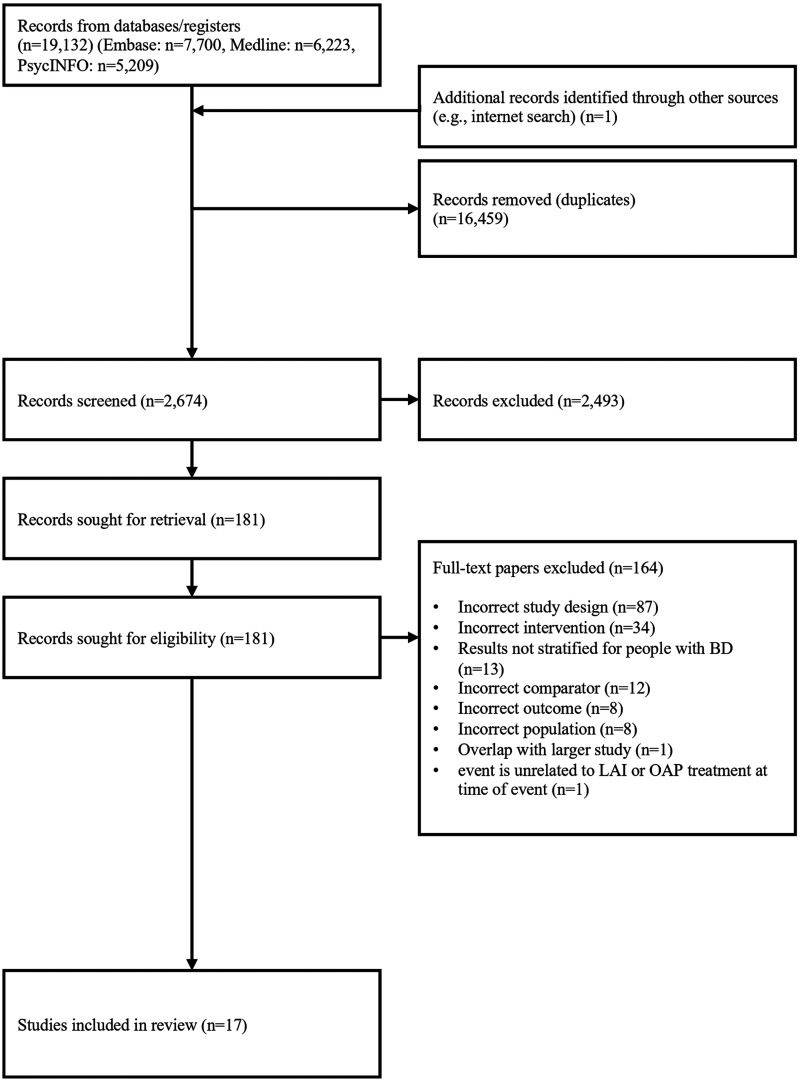
PRISMA flow diagram.

After duplicate removal, 2,674 records were screened for their titles and abstracts from three electronic databases. Altogether, 2,493 records were excluded after screening titles and abstracts. The full texts of 181 records were reviewed, and 164 were excluded not meeting inclusion criteria (Supplementary Table S1). The primary reason for exclusion at this phase was recorded as per the pre-specified hierarchical criteria for exclusion. Ultimately, 17 studies met all inclusion criteria,^19,20,22,^^29–41^ including one study which was identified on a pre-print server (Figure 1).^21^

Altogether, four cohort studies were included in quantitative analyses, which encompassed 6,186 participants with BD, with a weighted mean proportion of 50.4% females, a weighted mean age of 42.2 years and a mean follow-up period of 2.4 years (Table 1). Studies were conducted in Taiwan (*n* = 2), Turkey (*n* = 1) and Finland (*n* = 1). Three out of four studies (75%) were rated as having “good” quality and one study (25%) reached a NOS rating of <7.

**Table 1.** List of Included Studies Assessing the Impact of Antipsychotic Long-Acting Injectable Formulation in People With Bipolar Disorder.

Author and Year, Country	Setting	Total N	NOS	Adjusted estimates	Number of adjusted factors	% female	Mean age (years)	Mean follow-up period (years)	LAI type	OAP comparator	(Co-)Primary outcome(s)
**Mirror-image studies**											
Bartoli 2022^29^, Italy	Community and hospital	68	9	no	NA	51.5	45.7	1	Mixed LAI (ARI, PAL, RIS, HAL, FLU)	FGA + SGA	Mean number of hospitalisation days
Bartoli 2023^30^, Italy	Community and hospital	71	7	no	NA	46.5	46.2	1	Mixed LAI (ARI, PAL, RIS, HAL, FLU, ZUCLO)	FGA + SGA	Mean number of hospitalisation days
Caliskan 2020^31^, Turkey	Hospital	36	6	no	NA	25	38.9	1	PAL	Not specified	Mean number of hospitalisation days, number of hospitalisations
Chan 2016^32^, Taiwan	Community and hospital	77	6	no	NA	61	39	1	RIS	Not specified	Rate of ED visits
García-Carmona 2024^21^, Spain	Community and hospital	122	6	no	NA	NA	NA	2	Mixed LAI (PAL, ARI)	QUE, OLA, RIS	Mean number of hospitalisation days, number of hospitalisations
Goto 2023^20^, Japan	Community and hospital	39	6	no	NA	69.2	39.2	1	ARI	Not specified	Mean number of hospitalisation days
Hsieh 2017^33^, Taiwan	Community and hospital	287	7	no	NA	56.8	39.9	1	RIS	FGA + SGA	Number of ED visits
Littlejohn 1994^38^, UK	Community	18	6	no	NA	55	51	8.2	only FGA	only FGA (HAL, ZUCLO, FLU, PIP, FLUPH)	Mean number of hospitalisation days
Lähteenvuo 2018^36^, Finland	Community and hospital	NA	9	yes	NA	NA	NA	NA	Mixed LAI (RIS, PER, OLA, HAL, ZUCLO)	FGA + SGA	Psychiatric hospitalisation
Vieta 2008^39^, Spain	Hospital	29	5	no	NA	48.3	36.1	2	RIS	RIS, OLA, HAL	Number of hospitalisations
White 1993^40^, New Zealand	NA	16	6	no	NA	37.5	42	3.66	only FGA	Not specified	Mean number of mood episodes
Woo 2024^19^, South Korea	Hospital	75	6	no	NA	69.3	37.4	2	ARI	Not specified	Mean number of mood episodes
Yildizhan 2022^41^, Turkey	Community	17	7	no	NA	52.9	41.5	1	Mixed LAI (ARI, PAL, RIS)	Not specified	Mean number of hospitalisation days

		**Total N**	**Mean NOS ± SD**	**Adjusted estimates in %**	**Number of adjusted factors in %**	**Weighted mean % female**	**Mean age (weighted)**	**Mean (weighted) follow-up period (years)**	**% of LAI types**	**% of OAP type**	/
**Subtotal**	**Community + hospital: *k*** **=** **7 (53.8%);** **Hospital: *k*** **=** **3 (23.1%), Community: *k*** **=** **2 (15.4%), N/A: *k*** **=** **1 (7.7%)**	**3,676**	**Mean NOS, 6.6** **±** **1.2 (range 5-9)**	**No statistical adjustment: *k*** **=** **12 (92.3%); statistical adjustment: *k*** **=** **1 (7.7%)**	**All studies: N/A**	**53.5**	**45**.**4**	**5.9**	**Mixed LAIs: *k*** **=** **3 (23.1%).** **RIS-LAI: *k*** **=** **3 (23.1%). Mixed SGA-LAIs: *k*** **=** **2 (15.4%).** **Mixed FGA LAIs: *k*** **=** **2 (15.4%); ARI-LAI: *k*** **=** **2 (15.4%), PAL-LAI: *k*** **=** **1 (7.7%)**	**Mixed FGAs** **+** **SGAs: *k*** **=** **5 (38.5%);** **Not specified: *k*** **=** **6 (46.2%);** **Mixed SGAs: *k*** **=** **1 (7.7%); Mixed FGAs: *k*** **=** **1 (7.7%)**	

**Cohort studies**		**Total N**	**NOS**	**Adjusted estimates**	**Number of adjusted factors**	**% female**	**Mean age (years)**	**Mean follow-up period (years)**	**LAI type**	**OAP comparator**	
Korkmaz 2024^34^, Turkey	Hospital	116	6	no	NA	44	41.4	1	Mixed LAI (PAL, ARI, ZUCLO, RIS)	QUE, HAL, OLA, ARI	Time to relapse
Lin 2021^37^, Taiwan	Hospital	4,060	8	yes	6	49	42.4	1	Mixed LAI, (HAL, FLUP, RIS, PAL)	Not specified	Time to rehospitalisation
Lähteenvuo 2023^35^, Finland	Community and hospital	1,065	9	yes	14	56.4	41.7	9.3	Mixed LAI (OLA, HAL, ZUCLO, PAL, ARI, RIS, PER)	FGA + SGA	Psychiatric hospitalisation
Tien 2025^22^, Taiwan	Hospital	945	7	yes	10	NA	NA	1	FGA + SGA	Not specified	All-cause hospitalisation

		**Total N**	**Mean NOS ± SD**	**Adjusted estimates in %**	**Number of adjusted factors in %**	**Weighted mean % female**	**Mean age (weighted)**	**Mean (weighted) follow-up period (years)**	**% of LAI types**	**% of OAP type**	/
**Subtotal**	**Community + hospital: *k*** **=** **1 (25%);** **Hospital: *k*** **=** **3 (75%)**	**6,186**	**Mean NOS, 7.5** **±** **1.3 (range 6-9)**	**No statistical adjustment: *k*** **=** **1 (25%); statistical adjustment: *k*** **=** **1 (75%)**	**6 factors: *k*** **=** **1 (25%), 14 factors: *k*** **=** **1 (25%), 10 factors: *k*** **=** **1 (25%), N/A: *k*** **=** **1 (25%)**	**50.4**	**42**.**2**	**2.4**	**Mixed LAIs: *k*** **=** **4 (100%)**	**Mixed FGAs** **+** **SGAs: *k*** **=** **2 (50%);** **Not specified: *k*** **=** **2 (50%)**	

**Legend:** Cohort studies with within-individual comparisons were labelled as mirror-image studies for analyses.

Abbreviations: ARI = Aripiprazole, ED = emergency department; FGA = first-generation antipsychotic; FLU = Flupenthixol, FLUP = Fluphenazine, HAL = Haloperidol, LAI = long-acting injectable; NA = not available; NOS = Newcastle–Ottawa Scale; OLA = Olanzapine, PAL = Paliperidone, PER = Perphenazine, PIP = Pipothiazine, RIS = Risperidone, SD = standard deviation; SGA = second-generation antipsychotic; ZUCLO = zuclopenthixole.

Altogether, 13 mirror image studies were included in quantitative analyses, which encompassed 3,676 participants with BD, with a weighted mean proportion of 52.5% females, a weighted mean age of 45.4 years and a mean follow-up period of 5.9 years (Table 1). Studies were conducted in Italy (*n* = 2), Turkey (*n* = 2), Taiwan (*n* = 2), Spain (*n* = 2), Japan (*n* = 1), UK (*n* = 1), Finland (*n* = 1), New Zealand (*n* = 1) and South Korea (*n* = 1). Five studies (38.5%) were rated as “good” quality, and eight (61.5%) reached a NOS rating of <7.

### Covariates in Cohort Studies

Mean baseline BMI, proportion of people with rapid cycling BD, comorbid substance use disorder, personality disorder, anxiety and post-traumatic stress disorder (PTSD), the number of psychotropic medications, the number of previous suicide attempts, and the number previous mood episodes were not available in any of the four cohort studies.

Proportion of people with concomitant mood stabilisers was available in two out of four studies. Proportion of people with psychosis, duration of illness and number of previous admissions were available in one study. All-cause discontinuation estimates were available only in one cohort study.^34^ Mortality rates were not available from cohort studies.

### Primary Outcome

**Figure 2.**
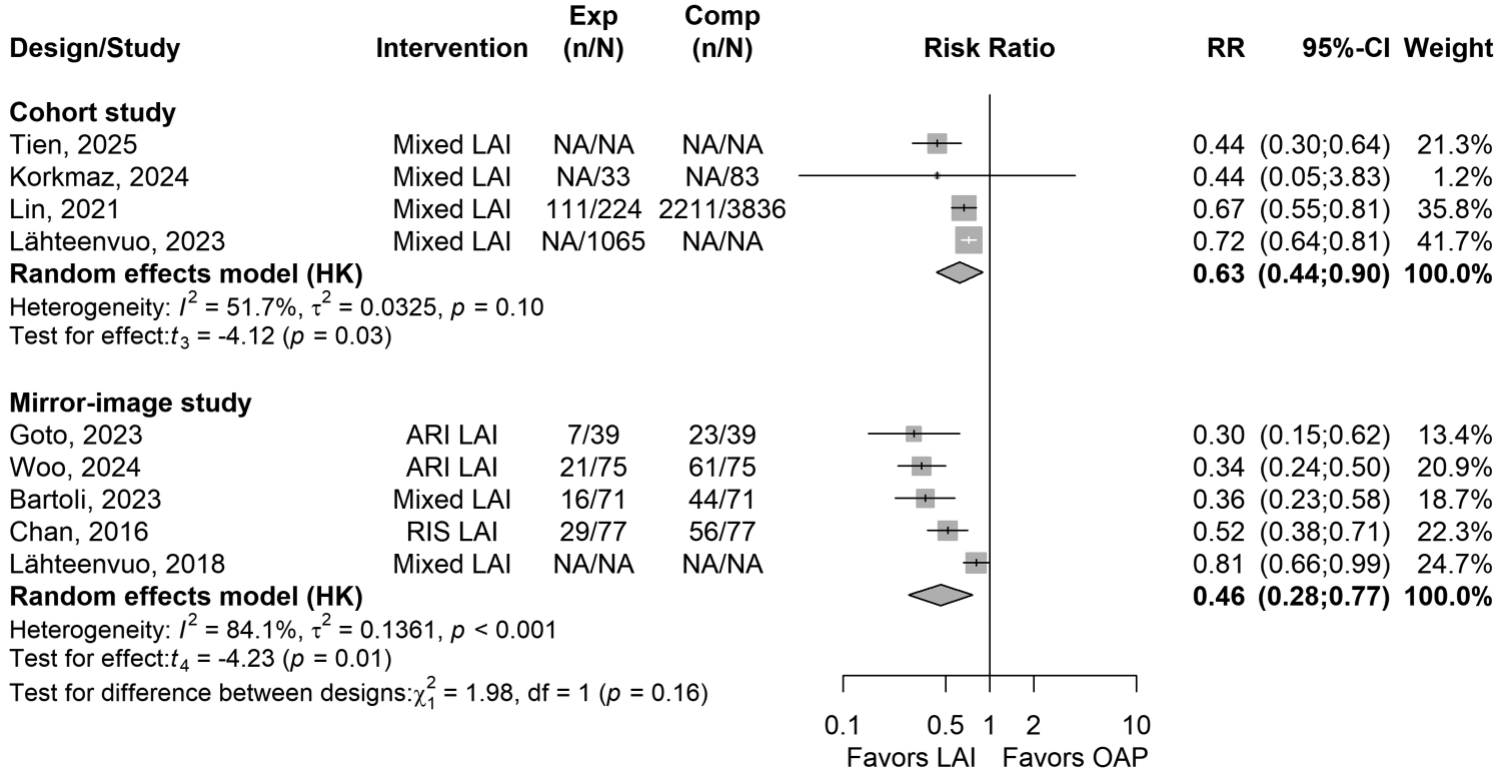
Forest plot of relative risk (RR) for combined psychiatric (re-)hospitalisation-relapse outcome (LAIs vs. OAP treatments) in people with BD in cohort (between-individual design) and in mirror-image studies (within-individual design). Abbreviations: AP = Antipsychotic, ARI = Aripiprazole, CI = Confidence Interval, LAI = Long-Acting Injectable, NOS = Newcastle–Ottawa Scale, RIS = Risperidone, RR = Relative Risk.

Overall, the RR for study-defined relapse (any hospitalisation or relapse) was significantly lower with LAIs as compared to (pre-)LAI OAP in cohort studies (*k* = 4, RR = 0.63, 95% CI = 0.44 to 0.90, *I*^2^ = 52%, *P* = 0.026) (Figure 2) and in mirror-image studies (*k* = 5, RR = 0.46, 95% CI = 0.28; 0.77, *I*^2^ = 84%, *P* = 0.013). The mean follow-up period was 13.5 ± 3.1 months in the cohort studies (*k* = 4) and 15.8 ± 5.5 months in the mirror-image studies (*k* = 5).

In sensitivity analyses, when only studies with a NOS rating of ≥7 were considered, LAIs were not significantly superior to OAPs anymore in cohort studies (*k* = 3, *P* = 0.78) and in mirror-image studies (*k* = 2, *P* = 0.38). When only cohort studies being adjusted for >5 factors were selected, results became significant again (*k* = 2, RR = 0.56, 95% CI = 0.37 to 0.84, *I*^2^ = 74%, *P* = 0.006).

When only second-generation antipsychotic LAIs were considered in mirror-image studies, LAIs were significantly superior compared to OAPs (*k* = 3, RR = 0.40, 95% CI = 0.20 to 0.80, *I*^2^ = 43%, *P* = 0.03), with significant superiority in each of these three individual second-generation antipsychotic LAI studies versus OAPs (aripiprazole-LAI: 2 studies, risperidone-LAI: 1 study) (all analyses shown in Supplementary Table 2).

## Key Secondary Outcomes

- Risk of (re-)hospitalisation and risk of relapse in cohort studies

Risk of hospitalisation due to mania was significantly lower with LAIs compared to OAPs over a follow-up period of 12 months in a single study (*k* = 1, RR = 0.44, 95% CI = 0.29–0.67, *P* < 0.001), while data regarding risk of hospitalisation due to depression were not available in cohort studies. Risk of psychiatric hospitalisation (*k* = 3, *P* = 0.078) was not significantly associated with LAI use versus OAP treatment with a mean follow-up period of 14 ± 3.5 months. Similarly, risk of non-psychiatric hospitalisation (*k* = 1, *P* = 0.23) and risk of any relapse (*k* = 1, *P* = 0.46) were not significantly associated with LAIs compared to OAP treatments in single studies with follow-up periods of 18.1 and 12 months, respectively.

(b) Risk of (re-)hospitalisation and risk of relapse in mirror-image studies

Risk of psychiatric hospitalisation was significantly decreased with LAIs compared to OAPs (*k* = 4, RR = 0.50, 95% CI = 0.25–0.99, *I*^2^ = 82%, *P* = 0.048), with a mean follow-up period of 13.8 ± 3.6 months.

The risk of hospitalisation due to depression (*k* = 2, RR = 0.46, 95% CI = 0.24; 0.86, *I*^2^ = 5%, *P* = 0.014) was significantly reduced with LAIs compared to pre-LAI OAP treatment, but hospitalisation due to mania was not (*k* = 2, *P* = 0.075). The mean follow-up for both outcomes was 12 ± 0 months.

The risk of any relapse was significantly reduced with LAIs compared to OAP in a single study (*k* = 1, RR = 0.34, 95% CI = 0.24–0.50, *P* < 0.001), as were the specific risks of relapse due to depression (*k* = 1, RR = 0.48, 95% CI = 0.27–0.86, *P* = 0.013) and due to mania (*k* = 1, RR = 0.26, 95% CI = 0.14–0.46, *P* < 0.001) with a mean follow-up of all three outcomes of 24 ± 0 months.

(c) Mean number of psychiatric hospitalisations in cohort studies

Evidence from a single study suggested that there was no effect of LAIs on the mean number of psychiatric hospitalisations compared to OAPs (*k* = 1, *P* = 0.39) during a mean follow-up period of 12 months.

(d) Mean number of psychiatric hospitalisations in mirror-image studies

**Figure 3.**
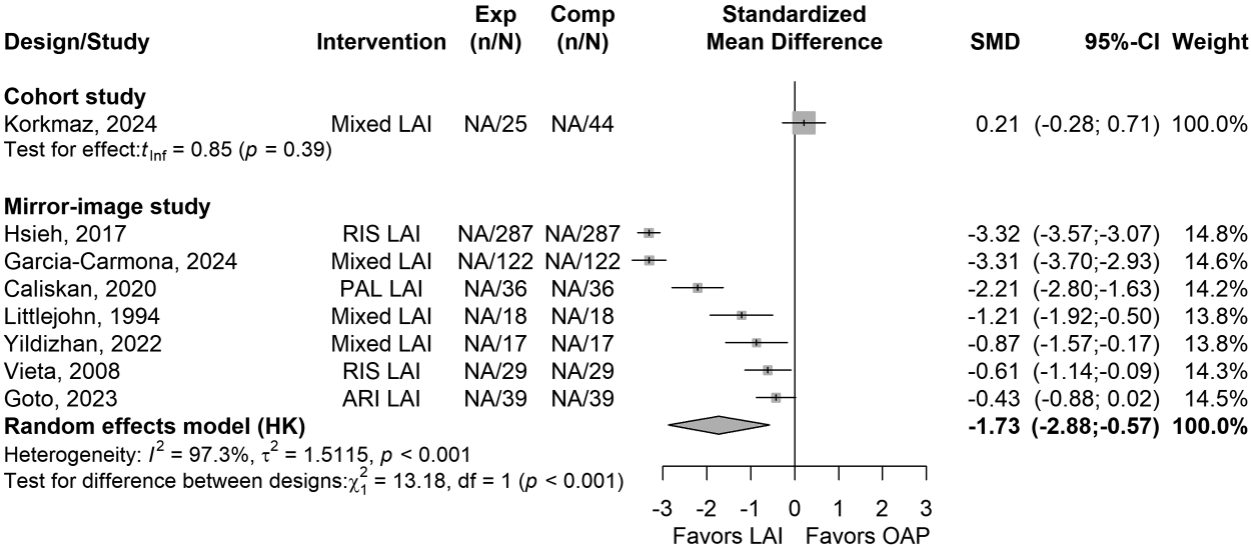
Forest plot of standardised mean difference (SMD) of mean number of psychiatric hospitalisations in mirror-image studies (LAIs vs. OAP) in people with BD. Abbreviations: AP = Antipsychotic, ARI = Aripiprazole, CI = Confidence Interval, LAI = Long-Acting Injectable, PAL = Paliperidone, RIS = Risperidone, SMD = Standardised Mean Difference.

Seven studies reported on the mean number of psychiatric hospitalisations. Overall, compared to the pre-LAI OAP treatments, LAIs were associated with fewer psychiatric hospitalisations (*k* = 7, SMD = −1.73, 95% CI = −2.88 to −0.57, *I*^2^ = 97%, *P* = 0.011, Figure 3), over a mean follow-up period of 27.8 ± 31.7 months (*k* = 7). Analyses for MDs are presented in the Supplementary Table S4.

**Figure 4.**
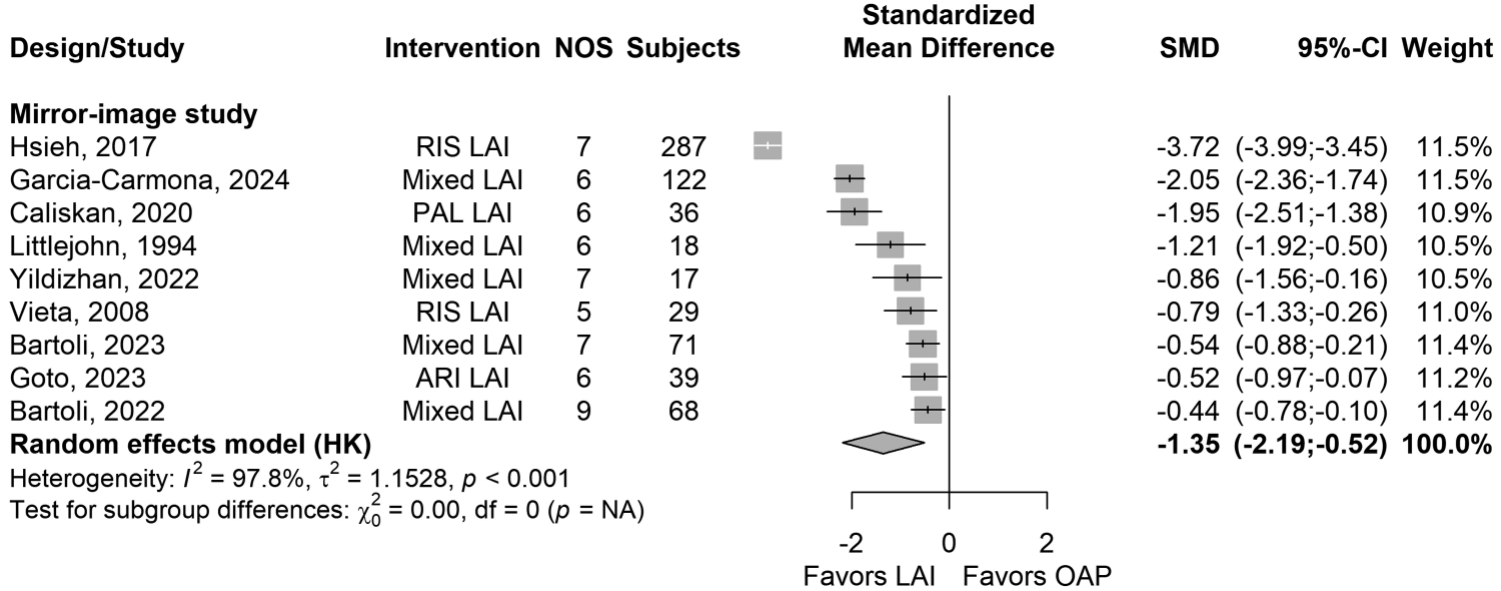
Forest plot of standardised mean difference (SMD) of mean days of hospitalisations in mirror-image studies (LAIs vs. OAP) in people with BD. Abbreviations: AP = Antipsychotic, ARI = Aripiprazole, CI = Confidence Interval, LAI = Long-Acting Injectable, NOS = Newcastle–Ottawa Scale, PAL = Paliperidone, RIS = Risperidone, SMD = Standardised Mean Difference.

The mean number of hospitalisations due to depression was not significantly associated with LAIs compared to OAPs (*k* = 3, *P* = 0.55). The mean number of hospitalisations due to mania was significantly lower with LAIs compared to pre-LAI OAP treatment (*k* = 3, SMD = −0.87, 95% CI = −1.19 to −0.56, *I*^2^ = 0%, *P* < 0.001). The mean follow-up period in both outcomes was 44.8 ± 46.8 months. Analyses for MDs are presented in the Supplementary Table S4.

(e) Mean days of hospitalisations in mirror-image studies

Nine studies reported on the mean number of days spent in psychiatric hospitals before and after LAI use. The mean follow-up period was 24.3 ± 28.3 months (*k* = 9). Significantly less days (*k* = 9, SMD = −1.35, 95% CI = −2.19 to −0.52, *I*^2^ = 98%, *P* = 0.006) were spent with LAIs compared to pre-LAI OAP treatment (Figure 4). The mean days of hospitalisation due to mania were significantly associated with LAI use (*k* = 2, SMD = −0.84, 95% CI = −1.43 to −0.25, *I*^2^ = 51%, *P* = 0.005), but the mean number of days of hospitalisation due to depression were not (*k* = 2, *P* = 0.23). For both outcomes, follow-up periods were 55.2 ± 61.1 months (*k* = 2). Analyses for MDs are presented in the Supplementary Table S4.

(f) Mean number of (any) mood episode(s) in cohort studies

Only one cohort study reported on the mean number of any mood episodes and showed no significant association with LAI use (*k* = 1, *P* = 1.0) during a mean follow-up period of 12 months.

(g) Mean number of (any) mood episode(s) in mirror-image studies

Four mirror-image design studies reported on the mean number of mood episodes before and after LAI use over a mean follow-up period of 23 ± 15.1 months. Compared to pre-LAI OAP treatments, LAI treatment was not significantly associated with the number of any mood episode (*k* = 4, *P* = 0.13). Also, during a mean follow-up period of 26.6 ± 16.1 months, the mean number of depressive mood episodes was not significantly associated with LAI use compared to OAP treatments (*k* = 3, *P* = 0.14). Only the mean number of manic mood episodes was significantly lower with LAI use compared to pre-LAI OAP use during the same follow-up period (*k* = 3, SMD = −1.13, 95% CI −2.00 to −0.26, *I*^2^ = 51%, *P* = 0.03). Analyses for MDs are presented in the Supplementary Table S4.

(h) Mean time to relapse in mirror-image studies

In only one mirror-image study with a mean follow-up of 24 months, risperidone-LAI treatment was associated with a significantly longer time to relapse compared with pre-LAI oral antipsychotic treatment (*k* = 1, SMD = −0.90, 95% CI = −1.44 to −0.36, *P* = 0.001 and MD = −265.4 days, 95% CI −417.03 to −113.77 days, *P* < 0.001).

### Other Secondary Outcomes

In a single cohort study, results for the mean number of ED visits were non-significant over the time period of 12 months (*k* = 1, *P* = 0.45). In mirror-image studies, the mean number of ED visits was not significantly lower among people receiving LAIs compared to pre-LAI OAP treatments during a mean follow-up period of 12 ± 0 months (*k* = 2, *P* = 0.092). Concerning the risk of ED visits, only one mirror-image study with a 12-month follow-up period was available and showed superior effectiveness of risperidone-LAI compared to OAP treatment (*k* = 1, RR = 0.39, 95% CI = 0.25 to 0.61, *P* < 0.001).

### Sensitivity Analysis, Publication Bias and GRADE Assessments

We were unable to conduct sensitivity analyses for all except the combined hospitalisation/relapse outcome and create a funnel plot as outlined in the protocol due to the small number of studies available for meta-analysis.

For cohort studies, the primary outcome results became non-significant when only adjusted cohort studies (*k* = 3, *P* = 0.78) or high-quality cohort studies (*k* = 3, *P* = 0.78) were taken into consideration.

For mirror-image studies, when only studies with SGA-LAIs were analysed, there were significantly lower study-defined relapse rates compared to pre-SGA-LAI OAPs (*k* = 3, RR = 0.40, 95% CI = 0.20–0.80, *I*^2^ = 43%, *P* = 0.03), but when only high-quality studies were selected, results were non-significant (*k* = 2, *P* = 0.38) (Supplementary Table S2).

Certainty of evidence in all reported outcomes was classified as *very low* or *low* (Supplementary Table S4). This classification aligns with the GRADE approach, which designates observational studies as starting at a “low” level of certainty by default. The downgrading to “very low” in certain instances reflects additional concerns, such as risk of bias, imprecision, inconsistency, or indirectness, as outlined in the GRADE Handbook.^24^

Sensitivity analyses contrasting the main mirror-image analysis with pre–post correlation coefficients of 0.3, 0.5 and 0.7 yielded similar results to the main analysis and are presented in Supplementary Tables S8 and S9.

## Discussion

This systematic review and meta-analysis included evidence from 17 observational studies regarding the effectiveness of LAIs in people with BD. In people with BD medication non-adherence is strongly associated with adverse clinical outcomes, including elevated risks of relapse, hospitalisation, suicide attempts and mortality.^42–45^ Furthermore, non-adherence substantially increases healthcare costs due to frequent hospitalisations.^43^ Historically, LAIs have sometimes but mostly only been used in clinical situations of non-adherence, which is as high as 60% in BD.^42^ However, recent expert consensus recommended to offer LAIs early in the disease course after the first manic episode in a shared decision-making process.^46,47^ This is a paradigm shift that has been discussed elsewhere.^46,48^ Given the lack of randomised controlled trials comparing LAIs with OAPs in patients with BD^14^ and potential selection factors in such trials biasing against showing benefits of LAIs over OAPs,^16^ a comprehensive meta-analysis of observational studies is essential to enhance statistical power and provide complementary evidence to inform treatment guidelines on the effectiveness of LAIs in people with BD. Our findings suggest a significant reduction in the study-defined risk of relapse/hospitalisation with LAIs versus OAPs in mirror-image (RR = 0.46) and cohort studies (RR = 0.63) but not for either study design when restricting the analyses to high-quality studies. Notably, when restricting mirror image studies to SGA-LAIs or when considering only cohort studies that adjusted for >5 factors, LAIs were significantly more effective than OAPs in reducing the study-defined risk of relapse. The latter finding demonstrates the importance of adequately adjusting for confounding variables in cohort studies to isolate the treatment effect and accurately assess its effectiveness.

In mirror-image studies, compared to the pre-LAI OAP treatments, LAI use was also associated with a significantly lower risk of psychiatric and depression-related hospitalisations and lower numbers of psychiatric and depression-related hospitalisations and hospitalisation days, and fewer manic episodes and mania-related hospitalisations. It has to be noted that the overall certainty of evidence in our findings is low or very low due to a limited number of high-quality observational studies; therefore, the current evidence does not (yet) support the routine use of LAIs for treating BD. Selection bias in cohort studies – where individuals with difficult-to-treat BD are more likely to receive LAIs – along with substantial variability in the type and number of covariates, confounded the estimates in our meta-analyses of cohort studies. Overall, more observational studies are needed to clarify whether the overall superiority of LAIs in mirror-image studies – which are less biased by confounding by indication – is driven by the prevention of mania. Nevertheless, this meta-analysis supports the recent expert recommendation to consider LAIs more often in the maintenance phase of BD.^46^

Moreover, our findings extend the ambiguous results of four RCTs each on SGA-LAIs in BD.^11,14^ The most recent meta-analysis indicated that across four RCTs (*n* = 929) SGA-LAIs (aripiprazole-LAI: *k* = 1; risperidone-LAI: *k* = 3) showed superiority over placebo for study-defined relapse (RR = 0.58, 95% CI = 0.49–0.68, *P* < 0.00001) and all-cause discontinuation (RR = 0.72, 95% CI = 0.64–0.82, *P* < 0.00001).^49^ However, SGA-LAIs did not differ in four studies, all with risperidone-LAI, (*n* = 394) from the active comparator groups (consisting of treatment as usual (TAU), TAU + OAPs, OAPs and oral olanzapine) regarding relapse rate (RR = 0.92, *P* = 0.79) and all-cause discontinuation (RR = 1.2, *P* = 0.31). Moreover, risperidone-LAI also did not differ from the mixed active control groups regarding mania-related relapse, mania symptoms and global illness severity, yet, the control group outperformed risperidone-LAI regarding depression-related relapse and depressive symptoms. Regarding adverse effects, the two groups did not differ on extrapyramidal side effects and weight gain, but active control interventions were associated with less prolactin-related AEs than risperidone-LAI. Nevertheless, as demonstrated in schizophrenia, LAIs may appear to be associated with greater risk of certain adverse effects when pooling all comparators together,^50^ but that difference disappeared for almost all adverse effects when the same antipsychotics in LAI and oral formulation were compared, with even reduced risk of the LAI versus OAP formulation,^51^ likely due to reduced peak-to-trough variations with LAIs.^52^ Taken together, the RCT evidence is mixed and insufficient regarding potential benefits of SGA-LAIs in the maintenance treatment of BD, and the evidence is restricted to risperidone-LAI, control groups were mixed and did not only contain OAPs, and the evidence is based on a small number of studies and participants.

Additionally, a recent meta-analysis focusing on the outcome after the discontinuation of LAIs or OAPs showed that people with BD who had received aripiprazole once-monthly before discontinuation appeared to have a lower recurrence rate after discontinuation than those who had received the oral formulation before discontinuation, with a number needed to treat that reached statistical significance at weeks 4 and 20.^15^ These data are corroborated by a meta-analysis of significantly lower relapse rates in people with schizophrenia after discontinuing LAIs versus OAPs (relapsed: 63.6% vs. 88.9%, number-needed-to-treat = 4, HR = 3.56, 95% CI = 2.68–4.27, *P* < 0.0001).^53^ Finally, the use of LAIs in the management of BD especially in people who showed partial or full remission of a manic episode with OAP treatment has been proposed in several guidelines.^54–56^ However, this recommendation was mainly made for risperidone-LAI.

Importantly, we were not able to disentangle the impact of combining LAIs with mood stabilisers. This is relevant as a recent network meta-analysis (NMA) indicated that the combination of an antipsychotic with a mood stabiliser appears to be the best option for the maintenance treatment in BD.^49,57^ However, this finding and recommendation was based on studies with OAPs only. Moreover, in meta-analyses olanzapine and quetiapine were especially effective – for quetiapine no LAI is available, and olanzapine LAI has a restricted use due to its risk for post-injection delirium/sedation syndrome and is rarely used due to relevant cardiometabolic effects.^58^ The latter may explain why none of the included studies used specifically olanzapine-LAI. However, as a subcutaneous olanzapine-LAI formulation without post-injection delirium/sedation syndrome risk is under development,^59^ this may introduce a further LAI treatment option in people with BD that has not been specifically evaluated here.

The findings on the potential benefits of LAIs compared to OAPs in mirror-image studies – particularly in preventing manic episodes – support the expert recommendation to offer LAIs regularly, including after the first manic episode.^46^ Our meta-analysis adds further evidence to the scientific discussion, where many clinicians, due to attitudinal factors or personal experience, often refrain from using LAIs – beyond practical concerns related to healthcare infrastructure, availability or regulatory approval.^46,60^ It should be noted that our findings need to be interpreted within the context of an evolving therapeutic landscape in BD, as novel extended-release formulations of risperidone^61^ and aripiprazole^62^ are broadening the scope for more targeted and individualised LAI treatment strategies.

Nevertheless, findings of this meta-analysis must be interpreted with caution considering the significant clinical and methodological heterogeneity.

First, the number of studies contributing data to the analyses of the primary outcome were small. Second, given the small sample size in some of the included studies, we suspect the presence of small-study effect which indicates greater likelihood of smaller studies in producing larger effects. Meta-regression analyses with sample size or baseline severity proxies, such as physical and psychiatric comorbidities could not be conducted, given no outcome analysis had at least ten studies. In this context, since LAI treatment might be especially beneficial for specific BD subgroups, we could for example not conduct meta-regression analysis on the observed estimates in relation to the proportion of people with a rapid cycling course in the study populations where LAI treatment is a viable option.^14^ More studies are needed to account for additional clinical factors such as comorbid substance use, and other mental comorbidities. Third, the NOS risk of bias tool is primarily used for cohort studies, however, in this review we also used it to examine mirror-image studies. Because the NOS does not account for methodological limitations specific to mirror-image designs – such as regression to the mean and temporal confounding – its ratings may overestimate the quality of these studies. Fourth, although observational studies have the advantage of providing real-world data, potential biases need to be taken into consideration. For example, there is a high likelihood of selection bias in cohort studies whereby generally people at higher risk for non-adherence and relapse/hospitalisation are selected for LAI use, which might have biased the results against a more robust effectiveness of LAIs. Conversely, in mirror-image studies, patients are often started on LAIs when they are in a particularly unstable clinical state, which can lead to regression to the mean in the post-mirror period, potentially inflating the benefits of LAIs. Fifth, the quantity and quality of covariates that were controlled for differed largely. This is especially relevant with regard to, for example, mood-stabilising treatments where information was lacking, so that we were not able to conduct meta-regression analyses on the impact of mood stabilisers on our reported outcomes. Sixth, unlike RCTs, the dose of LAIs and the baseline severity markers of people with BD were not comparable across studies, indicating further clinical heterogeneity. Seventh, between-study heterogeneity was high in the vast majority of our analyses. For instance, while five mirror-image studies demonstrated statistically significant effects individually, these effects became non-significant in sensitivity analyses of high-quality studies – likely due to the influence of random-effects modelling. Thus, this non-significant finding must be interpreted with caution. Eighth, methodological heterogeneity was present in the duration of follow-up periods, which also varied across both mirror-image and cohort studies and which could have influenced the results. We also acknowledge that the pooling of conventional mirror-image studies (e.g., oral-to-LAI comparisons) with the two studies by Lähteenvuo et al.,^35,36^ which accounted for temporal order of antipsychotic medication, may introduce an additional source of heterogeneity. Finally, it was not possible to generate pooled data on tolerability outcomes or all-cause discontinuation in mirror image or cohort studies and on mortality in cohort studies. Despite all of these limitations, this systematic review and meta-analysis summarises the currently available evidence regarding the effectiveness of LAIs for BD maintenance treatment in naturalistic settings.

## Conclusion

Overall, this systematic review and meta-analysis suggests that LAIs, compared to OAP treatments, may be effective in improving study-defined relapse/hospitalisation rate, as well as the number and mean days of hospitalisations in BD. The vast majority of our findings point towards the same direction of a superiority of LAIs in the maintenance treatment of people with BD but in the absence of tolerability and all-cause discontinuation data, our findings should be interpreted with caution. Future studies are needed that assess the effectiveness and tolerability of LAIs using pragmatic trials, which will allow for longer participant follow-up, less residual confounding and more accurate estimates. These results will be able to further inform clinical practice and guidelines. Until then, the current data, paired with information from RCTs in people with BD,^11,14^ related data from schizophrenia^50^ and expert consensus statements^46,47^ may indicate the potential utility of LAIs in the maintenance treatment of BD and that clinicians should at least inform patients about this option and offer them a potential trial with a LAI.

## Supplemental Material

sj-docx-1-cpa-10.1177_07067437251412576 - Supplemental material for Effectiveness of Long-Acting Injectable Antipsychotics Versus Oral Antipsychotics in People With Bipolar Disorder: A Systematic Review and Meta-Analysis of Observational Studies: Efficacité des antipsychotiques injectables à action prolongée par rapport aux antipsychotiques oraux chez les personnes atteintes de troubles bipolaires : revue systématique et méta-analyse d'études observationnelles Supplemental material, sj-docx-1-cpa-10.1177_07067437251412576 for Effectiveness of Long-Acting Injectable Antipsychotics Versus Oral Antipsychotics in People With Bipolar Disorder: A Systematic Review and Meta-Analysis of Observational Studies: Efficacité des antipsychotiques injectables à action prolongée par rapport aux antipsychotiques oraux chez les personnes atteintes de troubles bipolaires : revue systématique et méta-analyse d'études observationnelles by Elias Wagner, Saguna Katyal, In Ok Lee, Sabah Tasnim, Hajar El Wadia, Matin Mortazavi, Juan Antonio García-Carmona, Alkomiet Hasan, Ian Colman, Heidi Taipale, Jari Tiihonen, Christoph U Correll, Mikkel Højlund and Marco Solmi in The Canadian Journal of Psychiatry
