# Supplementary material for: Effectiveness of Long-Acting Injectable Antipsychotics Versus Oral Antipsychotics in People With Bipolar Disorder: A Systematic Review and Meta-Analysis of Observational Studies: Efficacité des antipsychotiques injectables à action prolongée par rapport aux antipsychotiques oraux chez les personnes atteintes de troubles bipolaires : revue systématique et méta-analyse d'études observationnelles
Source: Can J Psychiatry. 2026 Jan 30:07067437251412576. Online ahead of print. doi: 10.1177/07067437251412576 (PMC12858392; doi:10.1177/07067437251412576)
Supplement: sj-docx-1-cpa-10.1177_07067437251412576 - Supplemental material for Effectiveness of Long-Acting Injectable Antipsychotics Versus Oral Antipsychotics in People With Bipolar Disorder: A Systematic Review and Meta-Analysis of Observational Studies: Efficacité des antipsychotiques injectables à action  [file sj-docx-1-cpa-10.1177_07067437251412576.docx]

**Supplementary Material**

**Effectiveness of Long-Acting Injectable Antipsychotics versus Oral Antipsychotics in People with Bipolar Disorder: A Systematic Review and Meta-Analysis of Observational Studies.**

**Supplementary Methods**

**Supplementary Box 1.** Literature search strategy.

The search strategy was peer-reviewed by a librarian at The Ottawa Hospital as per the guidelines in the Cochrane Handbook for Systematic Reviews of Interventions.

**Ovid MEDLINE(R) ALL <inception to March 25, 2025>**
1     bipolar disorder/ 47192
2     (bipolar adj3 (disorder* or affective* or ill* or psycho* or depress* or mood or mania)).ti,ab,kf.    47532
3     (manic adj3 (disorder* or depress*)).ti,ab,kf.  7225
4     (maniodepressi* or "mano depressi*").ti,ab,kf.  3
5     1 or 2 or 3 or 4  66395
6     Delayed-Action Preparations/  42415
7     (("Delayed action" or "long-acting" or month* or depot* or intramusc* or "intra-musc*" or "slow release" or "delayed release" or "prolonged-action" or "controlled-release") adj3 (prep* or inject* or formula* or therap* or drug* or medication* or administration* or dose* or dosage* or treatment* or effect*)).ti,ab,kf.      218973
8     (injections/ or injections, Intramuscular/) and ("Delayed action" or "long-acting" or month* or depot* or intramusc* or "intra-musc*" or "slow release" or "delayed release" or "prolonged-action" or "controlled-release").ti,ab,kf.     24482
9     6 or 7 or 8 261846
10    5 and 9     1072

**Embase Classic+Embase <inception to 2025 March 25>**
1     bipolar disorder/ 88337
2     (bipolar adj3 (disorder* or affective* or ill* or psycho* or depress* or mood or mania)).ti,ab,kf.    73407
3     (manic adj3 (disorder* or depress*)).ti,ab,kf.  10193
4     (maniodepressi* or "mano depressi*").ti,ab,kf.  10
5     1 or 2 or 3 or 4  109257
6     delayed release formulation/  9468
7     (("delayed release" or "long-acting" or month* or depot* or intramusc* or "intra-musc*" or "slow release" or "delayed-action" or "prolonged-action" or "controlled-release") adj3 (prep* or inject* or formula* or therap* or drug* or medication* or administration* or dose* or dosage* or treatment* or effect*)).ti,ab,kf.      367002
8     injection/ and ("delayed release" or "long-acting" or month* or depot* or intramusc* or "intra-musc*" or "slow release" or "delayed-action" or "prolonged-action" or "controlled-release").ti,ab,kf.  38355
9     intramuscular drug administration/ and ("delayed release" or "long-acting" or month* or depot* or inject* or "slow release" or "delayed-action" or "prolonged-action" or "controlled-release").ti,ab,kf.    25623
10    6 or 7 or 8 or 9  410686
11    5 and 10    1912

**APA PsycInfo <inception to March 25 2025>**
1     bipolar disorder/ 32553
2     bipolar I disorder/     1556
3     bipolar II disorder/    797
4     (bipolar adj3 (disorder* or affective* or ill* or psycho* or depress* or mood or mania)).ti,ab. 40362
5     (manic adj3 (disorder* or depress*)).ti,ab.     7739
6     (maniodepressi* or "mano depressi*").ti,ab.     1
7     (("Delayed action" or "long-acting" or month* or depot* or intramusc* or "intra-musc*" or "slow release" or "delayed release" or "prolonged-action" or "controlled-release") adj3 (prep* or inject* or formula* or therap* or drug* or medication* or administration* or dose* or dosage* or treatment* or effect*)).ti,ab.   19891
8     injections/ and ("Delayed action" or "long-acting" or month* or depot* or intramusc* or "intra-musc*" or "slow release" or "delayed release" or "prolonged-action" or "controlled-release").ti,ab.    1048
9     intramuscular injections/ and ("Delayed action" or "long-acting" or month* or depot* or intramusc* or "intra-musc*" or "slow release" or "delayed release" or "prolonged-action" or "controlled-release").ti,ab.  272
10    1 or 2 or 3 or 4 or 5 or 6    47386
11    7 or 8 or 9 20353
12    10 and 11   571

**Supplementary Table 1.** List of excluded studies

**Supplementary Table 1.** List of excluded studies.

| **Author and year** | **Reason for exclusion** |
| --- | --- |
| Kong 2023^1^ | Incorrect intervention |
| Vgontzas 2024^2^ | Results not stratified for BD patients |
| Harlin 2023^3^ | Incorrect study design |
| Bartoli 2023^4^ | Incorrect study design |
| Jing 2023^5^ | Incorrect intervention |
| Youn 2022^6^ | Incorrect intervention |
| Ibrahim 2021^7^ | Incorrect comparator |
| McVoy 2023^8^ | Incorrect study design |
| Perkins 2023^9^ | Incorrect intervention |
| Tidmore 2022^10^ | Results not stratified for BD patients |
| Bedggood 2022^11^ | Results not stratified for BD patients |
| Kan 2022^12^ | Incorrect intervention |
| Correll 2022^13^ | Incorrect study design |
| Kadakia 2021^14^ | Incorrect intervention |
| Consoloni 2021^15^ | Incorrect study design |
| Koh 2021^16^ | Incorrect study design |
| Garcia-Carmona 2020^17^ | Incorrect comparator |
| Tournier 2019^18^ | Incorrect intervention |
| Kishi 2019^19^ | Incorrect study design |
| Ng-Mak 2019^20^ | Incorrect intervention |
| Yan 2018^21^ | Incorrect outcome |
| Yan 2018^22^ | Incorrect comparator |
| Nestsiarovich 2018^23^ | Incorrect intervention |
| Maestri 2018^24^ | Results not stratified for BD patients |
| Aggarwal 2018^25^ | Incorrect study design |
| Yan 2018^26^ | Incorrect comparator |
| Greene 2018^27^ | Incorrect outcome |
| Ceylan 2017^28^ | Incorrect comparator |
| Bjorklund 2017^29^ | Incorrect intervention |
| Nikolic 2017^30^ | Results not stratified for BD patients |
| Aguilar 2019^31^ | Results not stratified for BD patients |
| Clinebell 2017^32^ | Incorrect study design |
| Molina 2016^33^ | Incorrect intervention |
| Bonafede 2015^34^ | Incorrect intervention |
| Karamustafalioglu 2014^35^ | Incorrect intervention |
| Locklear 2014^36^ | Incorrect intervention |
| Taylor 2014^37^ | Incorrect comparator |
| Locklear 2013^38^ | Incorrect intervention |
| Heesch 2016^39^ | Results not stratified for BD patients |
| Boarati 2013^40^ | Incorrect outcome |
| Gigante 2012^41^ | Incorrect study design |
| Pillarella 2012^42^ | Incorrect intervention |
| Cleary 2012^43^ | Incorrect study design |
| Perrin 2012^44^ | Incorrect population |
| Tang 2010^45^ | Incorrect intervention |
| Rais 2010^46^ | Incorrect population |
| Peuskens 2007^47^ | Incorrect study design |
| Macfadden 2011^48^ | Incorrect study design |
| Malempati 2011^49^ | Incorrect study design |
| Wang 2011^50^ | Incorrect intervention |
| Wilder 2010^51^ | Incorrect study design |
| Quiroz 2010^52^ | Incorrect study design |
| Benabarre 2009^53^ | Incorrect outcome |
| Yatham 2009^54^ | Incorrect study design |
| Castle 2009^55^ | Incorrect population |
| Citrome 2009^56^ | Incorrect intervention |
| Keith 2009^57^ | Incorrect study design |
| Gianfrancesco 2008^58^ | Incorrect intervention |
| Franks 2008^59^ | Incorrect study design |
| Sanford 2008^60^ | Incorrect study design |
| Malempati 2008^61^ | Incorrect study design |
| El-Mallakh 2007^62^ | Incorrect study design |
| Fu 2006^63^ | Incorrect intervention |
| Schimmelmann 2005^64^ | Incorrect comparator |
| Anonymous 2005^65^ | Incorrect intervention |
| Kusumakar 2002^66^ | Incorrect study design |
| Durbano 2002^67^ | Incorrect study design |
| Goodnick 1998^68^ | Incorrect study design |
| Negron 1996^69^ | Incorrect intervention |
| Kenar 2023^70^ | Incorrect population |
| Martinotti 2020^71^ | Incorrect study design |
| Pappa 2020^72^ | Results not stratified for BD patients |
| Mace 2018^73^ | Results not stratified for BD patients |
| Ziblak 2019^74^ | Incorrect study design |
| Yavuz 2023^75^ | Incorrect study design |
| Aguglia 2022^76^ | Incorrect outcome |
| Mora Cortes 2023^77^ | Incorrect study design |
| Pappa 2023^78^ | Results not stratified for BD patients |
| Hodson 2022^79^ | Incorrect study design |
| Veyej 2022^80^ | Incorrect outcome |
| Nguyen 2022^81^ | Incorrect comparator |
| Doolabh 2022^82^ | Incorrect study design |
| Lahteenvuo 2022^83^ | Incorrect study design |
| Yavuz 2022^84^ | Incorrect study design |
| Carmassi 2021^85^ | Results not stratified for BD patients |
| Fan 2021^86^ | Incorrect intervention |
| Medinas 2021^87^ | Incorrect study design |
| Barea 2021^88^ | Incorrect study design |
| Tringali 2021^89^ | Incorrect study design |
| Bernal 2021^90^ | Incorrect study design |
| Liu 2021^91^ | Incorrect study design |
| Perez Da Silva 2020^92^ | Incorrect intervention |
| Pappa 2020^93^ | Incorrect study design |
| Cirnigliaro 2020^94^ | Incorrect study design |
| Pappa 2020^95^ | Incorrect study design |
| Riedford 2020^96^ | Incorrect study design |
| Shere 2020^97^ | Incorrect study design |
| Pappa 2019^98^ | Results not stratified for BD patients |
| Florentino 2019^99^ | Incorrect study design |
| Inci Kenar 2019^100^ | Incorrect study design |
| Brito Santana 2019^101^ | Incorrect population |
| Iglesias 2019^102^ | Incorrect study design |
| Vieta 2019^103^ | Incorrect study design |
| Campos 2019^104^ | Incorrect study design |
| Janzen 2018^105^ | Incorrect population |
| Greene 2018^106^ | Incorrect study design |
| Pereira Sanchez 2018^107^ | Incorrect study design |
| Munoz Martinez 2018^108^ | Incorrect study design |
| Monteagudo 2018^109^ | Incorrect study design |
| Greene 2017^110^ | Incorrect comparator |
| Yan 2017^111^ | Incorrect outcome |
| Greene 2017^112^ | Incorrect population |
| Yan 2017^113^ | Incorrect study design |
| Fernandez-Miranda 2017^114^ | Incorrect intervention |
| Vannini 2017^115^ | Incorrect study design |
| Sajatovic 2016^116^ | Incorrect study design |
| Maestri 2016^117^ | Incorrect study design |
| Secchi 2016^118^ | Incorrect study design |
| Tsopelas 2016^119^ | Incorrect study design |
| Pascual 2016^120^ | Incorrect study design |
| Fernandez-Quintana 2016^121^ | Incorrect study design |
| Tournier 2016^122^ | Incorrect study design |
| Perez Lopez 2016^123^ | Incorrect study design |
| Lin 2016^124^ | Incorrect study design |
| Ceskova 2015^125^ | Incorrect study design |
| Zoltan 2015^126^ | Incorrect intervention |
| Obrocea 2015^127^ | Incorrect study design |
| Reinstatler 2015^128^ | Incorrect study design |
| Modesitt 2015^129^ | Incorrect comparator |
| Hsieh 2015^130^ | Incorrect study design |
| Ketter 2015^131^ | Incorrect study design |
| Lin 2015^132^ | Incorrect study design |
| Ostinelli 2015^133^ | Incorrect intervention |
| Seetasith 2014^134^ | Incorrect study design |
| Yen 2014^135^ | Incorrect comparator |
| Patel 2013^136^ | Results not stratified for BD patients |
| Rossi 2012^137^ | Incorrect study design |
| Cutts 2012^138^ | Incorrect study design |
| Pandey 2012^139^ | Incorrect study design |
| Augsten 2012^140^ | Incorrect study design |
| Locklear 2012^141^ | Incorrect study design |
| Jhawar 2011^142^ | Incorrect study design |
| Gouker 2011^143^ | Incorrect study design |
| Locklear 2011^144^ | Incorrect intervention |
| Campos Mangas 2011^145^ | Incorrect study design |
| Leotsakou 2009^146^ | Incorrect intervention |
| Morrato 2009^147^ | Incorrect intervention |
| Pelayo-Teran 2010^148^ | Incorrect study design |
| Svestka 2010^149^ | Incorrect study design |
| Carswell 2010^150^ | Incorrect population |
| Goswami 2009^151^ | Incorrect study design |
| Geddes 2009^152^ | Incorrect study design |
| Guerreiro 2009^153^ | Incorrect study design |
| Hassan 2009^154^ | Incorrect intervention |
| Hong 2009^155^ | Incorrect intervention |
| Nieto 2008^156^ | Incorrect study design |
| Ebrinc 2008^157^ | Incorrect comparator |
| Lage 2006^158^ | Incorrect intervention |
| Pandarakalam 2003^159^ | Incorrect study design |
| Samuel 2003^160^ | Incorrect outcome |
| Suzuki 2018^161^ | Incorrect study design |
| Mannion 1998^162^ | Incorrect study design |
| Holm 2022^163^ | Overlap with larger study population |
| Wingard 2017^164^ | event is unrelated to LAI or OAP treatment at time of event |

1. Kong L, Shen Y, Hu S, Lai J. The impact of quetiapine monotherapy or in combination with lithium on the thyroid function in patients with bipolar depression: A retrospective study. *CNS neuroscience & therapeutics* 2023; (101473265).

2. Vgontzas AN, Paschalidou A, Simos PG, et al. Impact of long-acting injectable antipsychotics vs. oral medication on relapses of patients with psychosis and bipolar disorder. *Psychiatry research* 2024; **332**: 115676.

3. Harlin M, Chepke C, Larsen F, et al. Aripiprazole Plasma Concentrations Delivered from Two 2-Month Long-Acting Injectable Formulations: An Indirect Comparison. *Neuropsychiatric disease and treatment* 2023; **19**: 1409-16.

4. Bartoli F, Cavaleri D, Nasti C, et al. Long-acting injectable antipsychotics for the treatment of bipolar disorder: evidence from mirror-image studies. *Therapeutic advances in psychopharmacology* 2023; **13**: 20451253231163682.

5. Jing P, Su J, Zheng C, Mei X, Zhang X. A retrospective study of psychotropic drug treatments in bipolar disorder at acute and maintenance episodes. *Frontiers in psychiatry* 2023; **14**: 1057780.

6. Youn H, Lee M-S, Jeong H-G, Kim S-H. Evaluation of factors associated with medication adherence in patients with bipolar disorder using a medication event monitoring system: a 6-month follow-up prospective study. *Annals of general psychiatry* 2022; **21**(1): 33.

7. Ibrahim HG, Malcolm BJ, Gogineni HP. Assessing Outcomes Between Risperidone Microspheres and Paliperidone Palmitate Long-Acting Injectable Antipsychotics Among Veterans. *Federal Practitioner* 2021; **38**(12): 586-91.

8. McVoy M, Levin JB. Updated strategies for the management of poor medication adherence in patients with bipolar disorder. *Expert review of neurotherapeutics* 2023; **23**(4): 365-76.

9. Perkins AJ, Khandker R, Overley A, et al. The impact of antipsychotic adherence on acute care utilization. *BMC psychiatry* 2023; **23**(1): 64.

10. Tidmore LM, Keast SL, Waters HC, Pareja KL, Cothran T, Skrepnek GH. Readmissions, costs, and duration to subsequent outpatient visit after hospital discharge among Medicaid beneficiaries utilizing oral versus long-acting injectable antipsychotics in bipolar disorder or schizophrenia. *Current medical research and opinion* 2022; **38**(9): 1621-30.

11. Bedggood M, Walton S, Bedggood M. Psychiatric hospitalisation before and after commencing long-acting injectable antipsychotic medication: a mirror-image study. *The New Zealand medical journal* 2022; **135**(1560): 37-47.

12. Kan ACO, Chan JKN, Wong CSM, Chen EYH, Chang WC. Psychotropic drug utilization patterns in pregnant women with bipolar disorder: A 16-year population-based cohort study. *European neuropsychopharmacology : the journal of the European College of Neuropsychopharmacology* 2022; **57**(bjh, 9111390): 75-85.

13. Correll CU, Chepke C, Gionfriddo P, et al. The post COVID-19 healthcare landscape and the use of long-acting injectable antipsychotics for individuals with schizophrenia and bipolar I disorder: the importance of an integrated collaborative-care approach. *BMC psychiatry* 2022; **22**(1): 32.

14. Kadakia A, Dembek C, Liu Y, Dieyi C, Williams GR. Hospitalization risk in pediatric patients with bipolar disorder treated with lurasidone vs. other oral atypical antipsychotics: a real-world retrospective claims database study. *Journal of medical economics* 2021; **24**(1): 1212-20.

15. Consoloni J-L, M'Bailara K, Perchec C, et al. Trajectories of medication adherence in patients with Bipolar Disorder along 2 years-follow-up. *Journal of affective disorders* 2021; **282**: 812-9.

16. Koh L-YC, Chandwani N, Lim S, Chan CYW. The concurrent use of two long-acting injectables in the maintenance treatment of bipolar disorder. *Bipolar disorders* 2021; **23**(3): 312-4.

17. Garcia-Carmona JA, Simal-Aguado J, Campos-Navarro MP, Valdivia-Munoz F, Galindo-Tovar A. Long-Acting Injectable Antipsychotics: Analysis of Prescription Patterns and Patient Characteristics in Mental Health from a Spanish Real-World Study. *Clinical drug investigation* 2020; **40**(5): 459-68.

18. Tournier M, Neumann A, Pambrun E, et al. Conventional mood stabilizers and/or second-generation antipsychotic drugs in bipolar disorders: A population-based comparison of risk of treatment failure. *Journal of affective disorders* 2019; **257**: 412-20.

19. Kishi T, Ikuta T, Sakuma K, Matsuda Y, Iwata N. Comparison of quetiapine immediate- and extended-release formulations for bipolar depression: A systematic review and network meta-analysis of double-blind, randomized placebo-controlled trials. *Journal of psychiatric research* 2019; **115**: 121-8.

20. Ng-Mak D, Halpern R, Rajagopalan K, Loebel A. Hospitalization risk in bipolar disorder patients treated with lurasidone versus other atypical antipsychotics. *Current medical research and opinion* 2019; **35**(2): 211-9.

21. Yan T, Greene M, Chang E, Hartry A, Touya M, Broder MS. All-cause hospitalization and associated costs in patients with schizophrenia or bipolar disorder initiating long-acting injectable antipsychotics. *Current medical research and opinion* 2018; **34**(1): 41-7.

22. Yan T, Greene M, Chang E, Hartry A, Touya M, Broder MS. Medication Adherence and Discontinuation of Aripiprazole Once-Monthly 400 mg (AOM 400) Versus Oral Antipsychotics in Patients with Schizophrenia or Bipolar I Disorder: A Real-World Study Using US Claims Data. *Advances in therapy* 2018; **35**(10): 1612-25.

23. Nestsiarovich A, Mazurie AJ, Hurwitz NG, et al. Comprehensive comparison of monotherapies for psychiatric hospitalization risk in bipolar disorders. *Bipolar disorders* 2018; **20**(8): 761-71.

24. Maestri TJ, Mican LM, Rozea H, Barner JC. Do Long-Acting Injectable Antipsychotics Prevent or Delay Hospital Readmission? *Psychopharmacology bulletin* 2018; **48**(3): 8-15.

25. Aggarwal A, Schrimpf L, Lauriello J. Aripiprazole Long-Acting Injectable for Maintenance Treatment of Bipolar I Disorder in Adults. *Clinical schizophrenia & related psychoses* 2018; **11**(4): 221-3.

26. Yan T, Greene M, Chang E, Touya M, Broder MS. Impact of initiating long-acting injectable antipsychotics on hospitalization in patients with bipolar I disorder. *Journal of comparative effectiveness research* 2018; **7**(11): 1083-93.

27. Greene M, Yan T, Chang E, Hartry A, Touya M, Broder MS. Medication adherence and discontinuation of long-acting injectable versus oral antipsychotics in patients with schizophrenia or bipolar disorder. *Journal of medical economics* 2018; **21**(2): 127-34.

28. Ceylan MF, Erdogan B, Tural Hesapcioglu S, Cop E. Effectiveness, Adverse Effects and Drug Compliance of Long-Acting Injectable Risperidone in Children and Adolescents. *Clinical drug investigation* 2017; **37**(10): 947-56.

29. Bjorklund LB, Horsdal HT, Mors O, Gasse C, Ostergaard SD. Psychopharmacological treatment of psychotic mania and psychotic bipolar depression compared to non-psychotic mania and non-psychotic bipolar depression. *Bipolar disorders* 2017; **19**(6): 505-12.

30. Nikolic N, Page N, Akram A, Khan M. The impact of paliperidone palmitate long-acting injection on hospital admissions in a mental health setting. *International clinical psychopharmacology* 2017; **32**(2): 95-102.

31. Aguilar M, Malcolm B. Effect of long-acting aripiprazole monohydrate on inpatient encounters: A retrospective mirror image study. *The mental health clinician* 2019; **9**(4): 258-62.

32. Clinebell K, Gannon J, Debrunner S, Roy Chengappa KN. Long-acting risperidone injections in a pregnant patient with bipolar disorder. *Bipolar disorders* 2017; **19**(7): 606-7.

33. Molina L, Recinos B, Paz B, et al. Factors Related to Early Clinical Effects of Quetiapine Extended-Release: A Multinational, Prospective, Observational Study. *Clinical drug investigation* 2016; **36**(6): 491-7.

34. Bonafede M, Locklear JC, Wahlqvist P, et al. Impact of once-daily extended-release quetiapine fumarate on hospitalization length in patients with acute bipolar mania. *Journal of comparative effectiveness research* 2015; **4**(1): 51-9.

35. Karamustafalioglu O, Reif A, Atmaca M, et al. Hospital stay in patients admitted for acute bipolar manic episodes prescribed quetiapine immediate or extended release: a retrospective non-interventional cohort study (HOME). *BMC psychiatry* 2014; **14**(100968559): 246.

36. Locklear JC, Wahlqvist P, Gustafsson U, Udd M, Fajutrao L, Eriksson H. Impact of extended-release quetiapine fumarate on hospitalization length and cost in schizophrenia and bipolar disorder patients: a retrospective, hospital-based, US-cohort analysis. *Journal of comparative effectiveness research* 2014; **3**(4): 335-44.

37. Taylor D, Olofinjana O. Long-acting paliperidone palmitate - interim results of an observational study of its effect on hospitalization. *International clinical psychopharmacology* 2014; **29**(4): 229-34.

38. Locklear JC, Alemayehu B, Brody RS, et al. Treatment patterns, healthcare resource utilization and costs in patients with bipolar disorder, newly treated with extended release or immediate release quetiapine fumarate using US healthcare administrative claims data. *Clinical therapeutics* 2013; **35**(12): 1923-32.

39. Heesch CB, Moore TA, Gutierrez CA, Lee S. Hospitalizations and emergency room visits after initiation of long-acting injectable antipsychotics. *The mental health clinician* 2016; **6**(3): 134-41.

40. Boarati MA, Wang Y-P, Ferreira-Maia AP, Cavalcanti ARS, Fu-I L. Six-month open-label follow-up of risperidone long-acting injection use in pediatric bipolar disorder. *The primary care companion for CNS disorders* 2013; **15**(3).

41. Gigante AD, Lafer B, Yatham LN. Long-acting injectable antipsychotics for the maintenance treatment of bipolar disorder. *CNS drugs* 2012; **26**(5): 403-20.

42. Pillarella J, Higashi A, Alexander GC, Conti R. Trends in use of second-generation antipsychotics for treatment of bipolar disorder in the United States, 1998-2009. *Psychiatric services (Washington, DC)* 2012; **63**(1): 83-6.

43. Cleary A, Walsh F, Connolly H, et al. Monitoring and documentation of side effects from depot antipsychotic medication: an interdisciplinary audit of practice in a regional mental health service. *Journal of psychiatric and mental health nursing* 2012; **19**(5): 395-401.

44. Perrin E, Anand E, Dyachkova Y, Wagner T, Frediani S, Ballerini A. A prospective, observational study of the safety and effectiveness of intramuscular psychotropic treatment in acutely agitated patients with schizophrenia and bipolar mania. *European psychiatry : the journal of the Association of European Psychiatrists* 2012; **27**(4): 234-9.

45. Tang C-S, Yeh C-B, Huang Y-S, et al. Long-term effectiveness of aripiprazole in adolescents and young adults with bipolar disorder: A naturalistic study. *International journal of psychiatry in clinical practice* 2010; **14**(4): 252-6.

46. Rais AR, Williams K, Rais T, Singh T, Tamburrino M. Use of intramuscular ziprasidone for the control of acute psychosis or agitation in an inpatient geriatric population: an open-label study. *Psychiatry* 2010; **7**(1): 17-24.

47. Peuskens J, Kasper S, Arango C, et al. Management of acutely ill patients in the hospital setting: focus on quetiapine. *International journal of psychiatry in clinical practice* 2007; **11**(1): 61-72.

48. Macfadden W, Adler CM, Turkoz I, Haskins JT, Turner N, Alphs L. Adjunctive long-acting risperidone in patients with bipolar disorder who relapse frequently and have active mood symptoms. *BMC psychiatry* 2011; **11**: 171.

49. Malempati RN, Bond DJ, Kunz M, Malemati C, Cheng A, Yatham LN. Long-term efficacy of risperidone long-acting injectable in bipolar disorder with psychotic features: a prospective study of 3-year outcomes. *International clinical psychopharmacology* 2011; **26**(3): 146-50.

50. Wang Z, Kemp DE, Chan PK, et al. Comparisons of the tolerability and sensitivity of quetiapine-XR in the acute treatment of schizophrenia, bipolar mania, bipolar depression, major depressive disorder, and generalized anxiety disorder. *The international journal of neuropsychopharmacology* 2011; **14**(1): 131-42.

51. Wilder CM, Elbogen EB, Moser LL, Swanson JW, Swartz MS. Medication preferences and adherence among individuals with severe mental illness and psychiatric advance directives. *Psychiatric services* 2010; **61**(4): 380-5.

52. Quiroz JA, Yatham LN, Palumbo JM, Karcher K, Kushner S, Kusumakar V. Risperidone long-acting injectable monotherapy in the maintenance treatment of bipolar I disorder. *Biological psychiatry* 2010; **68**(2): 156-62.

53. Benabarre A, Castro P, Sanchez-Moreno J, et al. [Efficacy and safety of long-acting injectable risperidone in maintenance phase of bipolar and schizoaffective disorder]. *Actas espanolas de psiquiatria* 2009; **37**(3): 143-7.

54. Yatham LN, Kauer-Sant'Anna M, Bond DJ, Lam RW, Torres I. Course and outcome after the first manic episode in patients with bipolar disorder: prospective 12-month data from the Systematic Treatment Optimization Program For Early Mania project. *Canadian journal of psychiatry Revue canadienne de psychiatrie* 2009; **54**(2): 105-12.

55. Castle DJ, Udristoiu T, Kim CY, et al. Intramuscular olanzapine versus short-acting typical intramuscular antipsychotics: comparison of real-life effectiveness in the treatment of agitation. *The world journal of biological psychiatry : the official journal of the World Federation of Societies of Biological Psychiatry* 2009; **10**(1): 43-53.

56. Citrome L, Reist C, Palmer L, et al. Dose trends for second-generation antipsychotic treatment of schizophrenia and bipolar disorder. *Schizophrenia research* 2009; **108**(1-3): 238-44.

57. Keith S. Use of long-acting risperidone in psychiatric disorders: focus on efficacy, safety and cost-effectiveness. *Expert review of neurotherapeutics* 2009; **9**(1): 9-31.

58. Gianfrancesco FD, Sajatovic M, Rajagopalan K, Wang R-H. The association between treatment adherence and antipsychotic dose among individuals with bipolar disorder. *International clinical psychopharmacology* 2008; **23**(6): 305-16.

59. Franks MA, Macritchie KAN, Mahmood T, Young AH. Bouncing back: is the bipolar rebound phenomenon peculiar to lithium? A retrospective naturalistic study. *Journal of psychopharmacology* 2008; **22**(4): 452-6.

60. Sanford M, Scott LJ. Intramuscular aripiprazole : a review of its use in the management of agitation in schizophrenia and bipolar I disorder. *CNS drugs* 2008; **22**(4): 335-52.

61. Malempati RN, Bond DJ, Yatham LN. Depot risperidone in the outpatient management of bipolar disorder: a 2-year study of 10 patients. *International clinical psychopharmacology* 2008; **23**(2): 88-94.

62. El-Mallakh RS. Medication adherence and the use of long-acting antipsychotics in bipolar disorder. *Journal of psychiatric practice* 2007; **13**(2): 79-85.

63. Fu AZ, Christensen DB, Hansen RA, Liu GG. Second-generation antidepressant discontinuation and depressive relapse in adult patients with bipolar depression: results of a retrospective database analysis. *Clinical therapeutics* 2006; **28**(6): 979-89.

64. Schimmelmann BG, Conus P, Edwards J, McGorry PD, Lambert M. Diagnostic stability 18 months after treatment initiation for first-episode psychosis. *The Journal of clinical psychiatry* 2005; **66**(10): 1239-46.

65. Anonymous. Extended-release carbamazepine (Equetro) for bipolar disorder. *The Medical letter on drugs and therapeutics* 2005; **47**(1205): 27-8.

66. Kusumakar V. Antidepressants and antipsychotics in the long-term treatment of bipolar disorder. *Journal of Clinical Psychiatry* 2002; **63**(SUPPL. 10): 23-8.

67. Durbano F, Mencacci C, Dorigo D, Riva M, Buffa G. The long-term efficacy and tolerability of carbolithium once a day: an interim analysis at 6 months. *La Clinica terapeutica* 2002; **153**(3): 161-6.

68. Goodnick PJ, Dominguez RA, DeVane CL, Bowden CL. Bupropion slow-release response in depression: diagnosis and biochemistry. *Biological psychiatry* 1998; **44**(7): 629-32.

69. Negron AE, Leiderman EA, Parkadavil M, Cienfuegos A, Javitt DC. A naturalistic outcome study of risperidone treatment among hospital patients. *Psychiatric services (Washington, DC)* 1996; **47**(10): 1118-20.

70. Kenar ANI, Unal GA, Mert A, Ay AM. Potential benefits of combining two long-acting injectable antipsychotic: a retrospective study. *European Review for Medical and Pharmacological Sciences* 2023; **27**(18): 8609-13.

71. Martinotti G, Prestia D, Barbui C, et al. Reasons for initiating long-acting antipsychotics in psychiatric practice: findings from the STAR Network Depot Study. *Therapeutic Advances in Psychopharmacology* 2020; **10**:2045125320978102.

72. Pappa S, Barnett J, Mason K. P.850Real-world effectiveness of aripiprazole long-acting injectable. *European Neuropsychopharmacology* 2020; **40**(Supplement 1): S472.

73. Mace S, Dzahini O, O'Hagan M, Taylor D. Haloperidol decanoate long-acting injection (HDLAI): Results of a 1-year mirror-image study. *Therapeutic Advances in Psychopharmacology* 2018; **8**(9): 241-9.

74. Ziblak A, Kenar ANI. Long-acting injectable aripiprazole use in an inpatient sample from Turkey. *Psychiatry and Clinical Psychopharmacology* 2019; **29**(Supplement 1): 95-6.

75. Yavuz E, Altinbas K. Long-acting new generation antipsychotics in the maintenance treatment of bipolar disorders. *European Psychiatry* 2023; **66**(Supplement 1): S696.

76. Aguglia A, Fusar-Poli L, Natale A, et al. Factors Associated with Medication Adherence to Long-Acting Injectable Antipsychotics: Results from the STAR Network Depot Study. *Pharmacopsychiatry* 2022; **55**(6): 281-9.

77. Mora Cortes M, Gil-Sierra MD, Dominguez-Santana CM, Blanco Castano M, Cano Martinez G. Effectiveness and safety of different initial doses of aripiprazole intramuscular depot. *European Journal of Hospital Pharmacy* 2023; **30**(Supplement 1): A38-A9.

78. Pappa S, Barnett J, Mason K. A 10-Year Observational Study of the Use, Acceptability and Effectiveness of Long-Acting Paliperidone Palmitate: Implications for Clinical Decision Making. *CNS Drugs* 2023; **37**(1): 107-16.

79. Hodson N, Majid M, Vlaev I, Singh SP. Can incentives improve antipsychotic adherence in major mental illness? A mixed-methods systematic review. *BMJ Open* 2022; **12**(6): e059526.

80. Veyej N, Moosa MYH. Prescribing patterns of long-acting injectable antipsychotics in a community setting in South Africa. *South African Journal of Psychiatry* 2022; **28**:1809.

81. Nguyen T, Frayne J, Watson S, Lebedevs T, Teoh S, Galbally M. Long-acting injectable antipsychotic treatment during pregnancy: Outcomes for women at a tertiary maternity hospital. *Psychiatry Research* 2022; **313**: 114614.

82. Doolabh U, Yeap S. Examining long-acting injectable antipsychotic (depot) medication in the elderly: a five-year retrospective cross-sectional study evaluating depot use in an Australian psychogeriatric service. *Australasian Psychiatry* 2022; **30**(1): 31-6.

83. Lahteenvuo M, Paljarvi T, Tanskanen A, Taipale H, Tiihonen J. Comparative Effectiveness of Medications in Bipolar Disorder in Real-World Settings Based on 60,045 Patients. *Neuropsychopharmacology* 2022; **47**(Supplement 1): 261-2.

84. Yavuz E, Altinbas K. Long-acting new generation antipsychotics in the maintenance treatment of bipolar disorders. *European Psychiatry* 2022; **65**(Supplement 1): S404-S5.

85. Carmassi C, Milani F, Bertelloni CA, Massimetti E, Ceru A, Dell'Osso L. Comparing re-hospitalisation rates in a real-world naturalistic 24-month follow-up of psychotic patients with different treatment strategies: Oral versus LAI antipsychotics. *International Journal of Clinical Practice* 2021; **75**(3): e13787.

86. Fan Q, Huang H, Schmerold L, Dembek C, Dieyi C, Williams G. Healthcare resourse utilization and costs in patients with bipolar disorder treated with lurasidone or cariprazine: a retrospective analysis of insurance claims data. *Journal of Managed Care and Specialty Pharmacy* 2021; **27**(4-A SUPPL): S59-S60.

87. Medinas R, Caetano R, Quintao A, Azevedo F, Laginhas C. P.0407 Follow-up study on long-acting injectable antipsychotics on bipolar disorder at a portuguese inpatient psychiatric unit (Hospital Egas Moniz). *European Neuropsychopharmacology* 2021; **53**(Supplement 1): S295.

88. Barea MV, Melero PV, Castro CM, Solis MO, Espanol FV. P.0600 Evaluation of real clinical experience in outpatients with psychosis who undergo monthly and quarterly paliperidone palmitate treatments. *European Neuropsychopharmacology* 2021; **53**(Supplement 1): S440-S1.

89. Tringali AGM, Caldiroli A, Capuzzi E, Carnevali G, Clerici M, Buoli M. P.0767 Clinical and demographic predictors of long acting-injectable antipsychotic drugs discontinuation in subjects with psychotic disorders: a retrospective observational study. *European Neuropsychopharmacology* 2021; **53**(Supplement 1): S558-S9.

90. Bernal S, Forcada M, Gonzales LA, Ovejero S. P.0456 Early administration of aripiprazole long-acting formulations reduces length of hospital stay of psychiatric inpatients. *European Neuropsychopharmacology* 2021; **53**(Supplement 1): S333-S4.

91. Liu Y, Patterson ME, Sahil S, Stoner SC. PMH1 Examining ALL-Cause Admission Counts after Initial Antipsychotics Administration during Hospitalization for Schizophrenia, Schizoaffective Disorder, or Bipolar Disorder. *Value in Health* 2021; **24**(Supplement 1): S128.

92. Perez Da Silva C, Polonio Fuentes E, Hernandez Huerta D. Does long acting antipsychotic treatment reduce antipsychotic polypharmacy at discharge from hospitalization in psychiatric inpatient units? *European Psychiatry* 2020; **63**(Supplement 1): S701.

93. Pappa S, Mason K. Partial compliance with long-acting paliperidone palmitate and impact on hospitalization: a 6-year mirror-image study. *Therapeutic Advances in Psychopharmacology* 2020; **10**:2045125320924789.

94. Cirnigliaro G, Battini V, Invernizzi E, et al. P.465 From one-month to three-month paliperidone palmitate: an Italian real-world, retrospective, one-year mirror image study. *European Neuropsychopharmacology* 2020; **40**(Supplement 1): S263-S4.

95. Pappa S, Mason K, Barnett J. P.849A five-year follow up study of a naturalistic cohort treated with long-acting injectable antipsychotics. *European Neuropsychopharmacology* 2020; **40**(Supplement 1): S471.

96. Riedford K. Moderating perspectives of long acting injectable use of antipsychotics: A literature review. *CNS Spectrums* 2020; **25**(2): 289-90.

97. Shere S, Jacob P, Srinath S. Long acting injectable antipsychotic use in children and adolescents: A chart review. *Indian Journal of Psychiatry* 2020; **62**(7 Supplement 1): S48.

98. Pappa S, Mason K, Howard E. Long-Term effects of paliperidone palmitate on hospital stay and treatment continuation. *International Clinical Psychopharmacology* 2019; **34**(6): 305-11.

99. Florentino ISM, Junior JBD, Cantilino A. On the use of long-acting injectable aripiprazole in the treatment of bipolar disorder I. *Clinical Schizophrenia and Related Psychoses* 2019; **12**(4): 149-51.

100. Inci Kenar AN, Mert A, Matsar A. P.684 Potential benefit of polypharmacy of Long-acting injectable drugs: retrospective data from a psychiatry hospital. *European Neuropsychopharmacology* 2019; **29**(Supplement 6): S462-S3.

101. Brito Santana L, Spinola C, Neto D, Gago J. P.460 Duration of untreated psychosis and long acting antipsychotics prescription - two-year retrospective analysis of first-episode psychosis. *European Neuropsychopharmacology* 2019; **29**(Supplement 6): S326.

102. Iglesias RM, Arias DN, Quintana AF. A retrospective study of aripiprazole longacting once-monthly introduction patterns in galicia. *CNS Spectrums* 2019; **24**(1): 210.

103. Vieta E, Baker RA, Madera JJ, et al. Sustained functional recovery and symptom remission after maintenance treatment with aripiprazole once-monthly for patients with bipolar I disorder. *CNS Spectrums* 2019; **24**(1): 201-2.

104. Campos JA, Docasar L, Seoane AM. Use of palmitate of paliperidone 1-monthly in patients <40 years old: Results of real clinical practice. *European Neuropsychopharmacology* 2019; **29**(Supplement 1): S535.

105. Janzen D, Kuo IF, Leong C, Bolton J, Alessi-Severini S. Initiation of long-acting injectable antipsychotics in the Canadian province of Manitoba. *Pharmacoepidemiology and Drug Safety* 2018; **27**(Supplement 2): 321.

106. Greene M, Yan T, Chang E, Touya M. Impact of initiating long-acting injectables on all-cause and psychiatric hospitalization in patients with bipolar I disorder. *Value in Health* 2018; **21**(Supplement 1): S189.

107. Pereira Sanchez V, Arrieta M, Ferrer-Chinchilla N, et al. Retrospective follow-up data on the use of intramuscular paliperidone palmitate in adolescents and young adults. *European Psychiatry* 2018; **48**(Supplement 1): S382.

108. Munoz Martinez V, Asensio Aguerri L, Nuevo Fernandez L, Rodriguez Gomez-Carreno C, Mata Saenz B, Lopez Lavela E. Clinical use of long acting antipsychotic injection in an adolescente inpatient unit. *European Psychiatry* 2018; **48**(Supplement 1): S149.

109. Monteagudo E, Sanchez R, Castro JI, et al. Long-acting injectable aripiprazole: Sample of 26 outpatients treated with this antipsychotic in two community mental health services in Barcelona. *European Psychiatry* 2018; **48**(Supplement 1): S502-S3.

110. Greene M, Yan T, Chang E, Hartry A, Broder MS. Medication adherence and discontinuation in medicaid patients with dual diagnoses of schizophrenia and bipolar who initiated long acting injectable antipsychotic versus those who changed oral antipsychotics. *Value in Health* 2017; **20**(5): A22.

111. Yan T, Greene M, Chang E, Touya M, Broder MS. Medication adherence and discontinuation in patients with bipolar disorders who initiated a long acting injectable antipsychotic versus those who changed oral antipsychotics. *Value in Health* 2017; **20**(5): A301.

112. Greene M, Yan T, Stellhorn RA, Touya M, Broder MS. Comparisons of re-hospitalization rates in patients with bipolar disorder receiving long-acting injectable antipsychotics during hospitalization. *Value in Health* 2017; **20**(5): A302.

113. Yan T, Chang E, Hartry A, Broder M, Greene M. Hospitalizations and costs in bipolar disorder patients initiating long-acting injectable antipsychotics. *International Journal of Technology Assessment in Health Care* 2017; **33**(Supplement 1): 82-3.

114. Fernandez-Miranda JJ, Frias-Ortiz DF, Diaz-Fernandez S, Rubio-Rodriguez L. Bipolar type i and substance use disorders: Safety, tolerability and effectiveness of asenapine adjunctive treatment. *European Neuropsychopharmacology* 2017; **27**(Supplement 4): S816-S7.

115. Vannini C, Baroni G, Montemitro C, Martinotti G, Di Giannantonio M, Alessandrini M. Aripiprazole long-acting injection: A naturalistic study in a community setting. *European Neuropsychopharmacology* 2017; **27**(Supplement 4): S934.

116. Sajatovic M, Forester B, Tsai J, et al. Efficacy and safety of lurasidone in older adults with bipolar depression. *CNS Spectrums* 2016; **21**(1): 104.

117. Maestri TJ, Mican L, Rozea H, Goruganthu D, Barner J, Thach A. Do long-acting injectable antipsychotics prevent or delay hospital readmission? *Journal of Pharmacy Practice* 2016; **29**(3): 296.

118. Secchi A, Thomas T. An evaluation of patient involvement in the decision to initiate antipsychotic Long Acting Injections (LAIs) at Kent and Medway Social Care and Partnership Trust (KMPT). *International Journal of Pharmacy Practice* 2016; **24**(Supplement 3): 57-8.

119. Tsopelas C, Tzeferakos G, Kotsiouba I, Kalemi G, Douzenis A. Use of long acting injectables in mentally disordered offenders. *European Neuropsychopharmacology* 2016; **26**(Supplement 2): S520-S1.

120. Pascual JM, Mongil San Juan JM, Perez Revuelta J, Villagran Moreno JM. Use of long acting injectable antipsychotics in an acute adult psychiatric hospitalization unit. *European Neuropsychopharmacology* 2016; **26**(Supplement 2): S540-S1.

121. Fernandez-Quintana A, Vidal-Millares M, Garcia-Mahia MC. A 10 year follow-up study of long acting injectable risperidone use in severe mental disorders. *European Neuropsychopharmacology* 2016; **26**(Supplement 2): S563-S4.

122. Tournier M, Neumann A, Pambrun E, et al. Outcomes of three treatment strategies in bipolar disorder using conventional mood stabilizers and antipsychotic drugs. *Pharmacoepidemiology and Drug Safety* 2016; **25**(Supplement 3): 586-7.

123. Perez Lopez MD, Soto laguna M, Prados Gomez J, et al. Therapeutic attitudes and clinical global impression: A 2-year follow-up study of 33 outpatients with a mental disorder in treatment with paliperidone palmitate. *European Psychiatry* 2016; **33**(SUPPL.): S616-S7.

124. Lin SK, Chen WY. Comparison of efficacy and side effects of patients receiving long-acting injectable antipsychotics between schizophrenia and bipolar disorder. *Biological Psychiatry* 2016; **79**(9 SUPPL. 1): 405S.

125. Ceskova E. Current and future possibilities in the pharmacotherapy of bipolar disorder. *Ceska a Slovenska Psychiatrie* 2015; **111**(4): 185-8.

126. Zoltan R, Xenia G, Janos K. Treatment of bipolar depression with lamotrigine - rate of relapse and suicidal behaviour during 6 month follow-up treatment. *Neuropsychopharmacologia Hungarica* 2015; **17**(1): 7-13.

127. Obrocea GV, Abdijadid S, Vasan S, et al. Risperdal Consta is a cost-effective treatment: A 5-year retrospective study. *Asia-Pacific Psychiatry* 2015; **7**(Supplement 1): 19-20.

128. Reinstatler K, Gilmer L, Eby M, Wirick J. Readmissions in patients treated with longacting injectable antipsychotics during an acute psychiatric hospitalization. *Journal of Pharmacy Practice* 2015; **28**(3): 356.

129. Modesitt T, Kubascik E, Ott C. Evaluation of long-acting injectable (LAI) antipsychotics in children and adolescents within indiana medicaid. *Journal of Pharmacy Practice* 2015; **28**(3): 346.

130. Hsieh MH, Chuang PY, Wu CS, Chang CJ, Chung PF, Tang CH. Bipolar patients treated by long-acting injectable risperidone in Taiwan: A one-year mirror-image study using a national claims database. *Bipolar Disorders* 2015; **17**(SUPPL. 1): 135-6.

131. Ketter T, Tsai J, Silva R, Kroger H, Cucchiaro J, Loebel A. Lurasidone in the long-term treatment of patients with bipolar i disorder: Responder and remitter status during a 24-week open-label extension study. *European Neuropsychopharmacology* 2015; **25**(SUPPL. 2): S420-S1.

132. Lin SK, Cnhen WY. Comparison of subjective experiences between patients with schizophrenia and bipolar disorder receiving long-acting injectable antipsychotics. *Neuropsychopharmacology* 2015; **40**(SUPPL. 1): S203.

133. Ostinelli EG, Cavallotti S, Casetta C, Guanella E, D'Agostino A, Scarone S. Rapidity of action, effectiveness and adherence to treatment with asenapine: A real-world, observational study. *European Psychiatry* 2015; **30**(SUPPL. 1): 1127.

134. Seetasith A, Burudpakdee C. Comparison of resource use and health care costs in new initiators of long-acting injectable (LAI) and oral second generation antipsychotics. *Value in Health* 2014; **17**(7): A464.

135. Yen YC, Huang CY. Effectiveness of long-acting injectable antipsychotics in patients with bipolar I disorder. *Bipolar Disorders* 2014; **16**(SUPPL. 1): 97-8.

136. Patel MX, Matonhodze J, Baig MK, Taylor D, Szmukler G, David AS. Naturalistic outcomes of community treatment orders: Antipsychotic long-acting injections versus oral medication. *Journal of Psychopharmacology* 2013; **27**(7): 629-37.

137. Rossi A, Stratta P. Association of antipsychotics and mood stabilizers for treatment of mania. *Journal of Psychopathology* 2012; **18**(4): 389-96.

138. Cutts S, Gorsh B, Kostarides S, Makanji S, Makanji H, Lord T. Understanding reasons for nonadherence to atypical antipsychotic medications in claims data: Results from a pilot study. *Journal of Managed Care Pharmacy* 2012; **18**(7): 559.

139. Pandey A, Beezhold J, Pandey S, Hamer L. Paliperidone palmitate maintenance therapy: A quantitative review of patients. *European Neuropsychopharmacology* 2012; **22**(SUPPL. 2): S329.

140. Augsten A, McKee B, Locke CH, Camejo M. Comparison of psychiatric readmission rates of intramuscular paliperidone palmitate versus oral paliperidone. *Journal of Pharmacy Practice* 2012; **25**(2): 259.

141. Locklear JC, Alemayehu B, Brody RS, et al. A real-world us retrospective database analysis evaluating treatment patterns, health care resource utilization and costs in patients with bipolar disorder newly treated with immediate release (IR) or extended release (XR) quetiapine fumarate. *Value in Health* 2012; **15**(4): A83.

142. Jhawar A, Ruekert L, Lum C, Tristano S, Warney P. Comparison of long acting antipsychotic injections to the oral equivalents and the impact on recidivism. *Journal of Pharmacy Practice* 2011; **24**(2): 267.

143. Gouker M. Are providers documenting the efficacy of aripiprazole for adjunctive treatment of major depressive disorder after 3 months of treatment? *Journal of Pharmacy Practice* 2011; **24**(2): 264.

144. Locklear JC, Alemayehu B, Brody RS, et al. A US retrospective database analysis evaluating characteristics of patients with bipolar disorder prior to initiating treatment with immediate-release (IR) or extendedrelease (XR) quetiapine fumarate. *International Journal of Psychiatry in Clinical Practice* 2011; **15**(SUPPL. 2): 31.

145. Campos Mangas MC, Ruiz Feliu MA. Use of paliperidone in bipolar disorder. *European Psychiatry* 2011; **26**(SUPPL. 1).

146. Leotsakou C, Sardis A, Labiris C, et al. Paliperidone extended-release for the treatment of bipolar mania. *European Neuropsychopharmacology* 2009; **19**(SUPPL. 3): S489-S90.

147. Morrato EH, Cuffel B, Newcomer JW, Lombardo I, Kamat S, Barron J. Metabolic risk status and second-generation antipsychotic drug selection: A retrospective study of commercially insured patients. *Journal of Clinical Psychopharmacology* 2009; **29**(1): 26-32.

148. Pelayo-Teran JM, Torre OF, Trabajo-Vega PL, et al. Long acting antipsychotics: Comparison of first- and second-generation antipsychotic drugs in a community setting. *Schizophrenia Research* 2010; **117**(2-3): 494.

149. Svestka J, Hoschl C, Anders M, et al. Possibilities of aripiprazole use in the treatment of bipolar affective disorder expert consensus in the Czech Republic. *Psychiatrie* 2010; **14**(4): 218-24.

150. Carswell C, Wheeler A, Vanderpyl J, Robinson E. Comparative effectiveness of long-acting risperidone in New Zealand: A report of resource utilization and costs in a 12-month mirror-image analysis. *Clinical Drug Investigation* 2010; **30**(11): 777-87.

151. Goswami U, Rao H. Clinical and cost-effectiveness of risperidone long acting injection (RLAI) in the treatment of severe bipolar/schizoaffective disorders. *Bipolar Disorders* 2009; **11**(S1): 45-6.

152. Geddes J. BALANCE: Initial results and methodological aspects of trials of maintenance treatments. *Bipolar Disorders* 2009; **11**(S1): 6.

153. Guerreiro D, Navarro R, Telles D, et al. Psychiatric inpatient readmission in a general hospital: Preliminary report of a two years observational study. *European Psychiatry* 2009; **24**(SUPPL. 1): S793.

154. Hassan M, Lage MJ. Hospitalization rates among individuals with bipolar disorder initiating therapy with aripiprazole or quetiapine. *Value in Health* 2009; **12**(3): A187.

155. Hong J, Reed C, Novick D, Haro JM, Aguado J. Clinical and economic consequences of medication nonadherence in the treatment of patients with a manic/ mixed episode of bipolar disorder: Results from the european mania in bipolar longitudinal evaluation of medication (EMBLEM) study. *Value in Health* 2009; **12**(7): A355.

156. Nieto E, Villar L, Fernandez J, Hernandez C, Roman E, Bonet P. Evolution of psychotic inpatients treated with long-acting injectable risperidone after 1000 days of follow-up. *European Neuropsychopharmacology* 2008; **18**(S4): S421-S2.

157. Ebrinc S, Algul A, Ates MA, et al. Efficacy and safety of long-acting injectable risperidone during treatment of manic episode with non-compliance bipolar patients. *Neurology Psychiatry and Brain Research* 2008; **15**(1): 39-44.

158. Lage MJ, Rajagopalan K. Hospitalization and emergency department visits among patients treated with atypical antipsychotics: Evidence from a commercially insured population. *Journal of Applied Research* 2006; **6**(2): 115-25.

159. Pandarakalam JP. Atypical and conventional depot medications. *Hospital Medicine* 2003; **64**(11): 658-63.

160. Samuel M, Kisely S, Rhys-Gill E, et al. Switching patients to atypical oral antipsychotics: A retrospective audit of depot clinic attenders. *Journal of Mental Health* 2003; **12**(5): 513-9.

161. Suzuki H, Hibino H, Inoue Y, Mikami K. The advantages of introducing aripiprazole once-monthly 300 mg in the bipolar disorder-II maintenance treatment regime for 2 patients. *Asian Journal of Psychiatry* 2018; **34**:11.

162. Mannion L, Carney PA, Sloan D, Cody M. Depot antipsychotic drugs revisited. *Psychiatric Services* 1998; **49**(10): 1361-2.

163. Holm M, Tanskanen A, Lähteenvuo M, Tiihonen J, Taipale H. Comparative effectiveness of mood stabilizers and antipsychotics in the prevention of hospitalization after lithium discontinuation in bipolar disorder. *European Neuropsychopharmacol* 2022; **61**: 36-42.

164. Wingard L, Bodén R, Brandt L, Tiihonen J, Tanskanen A, Kieler H, Andersen M, Reutfors J. Reducing the rehospitalization risk after a manic episode: a population-based cohort of lithium, valproate, olanzapine, quetiapine and aripiprazole in monotherapy and combinations. *Journal of Affective Disorders* 2017; 217:16-23

**Supplementary Results**

**Supplementary Table 2.** Sensitivity analysis for the primary outcome any study-defined relapse (hospitalization-relapse).

| **Subgroup** | **k** | **RR (95% CI)** | **p-value** | **I^2^** |
| --- | --- | --- | --- | --- |
| Cohort studies | 4 | 0.63 (0.44; 0.90) | 0.026 | 52 |
| Mirror-image studies | 5 | 0.46 (0.28; 0.77) | 0.013 | 84 |
| Cohort NOS ≥7 | 3 | 0.63 (0.35; 1.14) | 0.78 | 67 |
| Mirror-image NOS ≥7 | 2 | 0.56 (0.00; 89.39) | 0.38 | 89 |
| Mirror SGA | 3 | 0.40 (0.20; 0.80) | 0.03 | 43 |
| Cohort adjusted | 3 | 0.63 (0.35; 1.14) | 0.78 | 67 |
| Cohort adjusted for >5 factors | 2 | 0.56 (0.37; 0.84) | 0.0055 | 74 |

Abbreviations: CI = confidence interval, k = number of studies, NOS = Newcastle-Ottawa Scale, RR = risk ratio, SGA = second-generation antipsychotic.

**Supplementary Figure 1.** Mean days of hospitalization – depression in mirror-image studies.

**
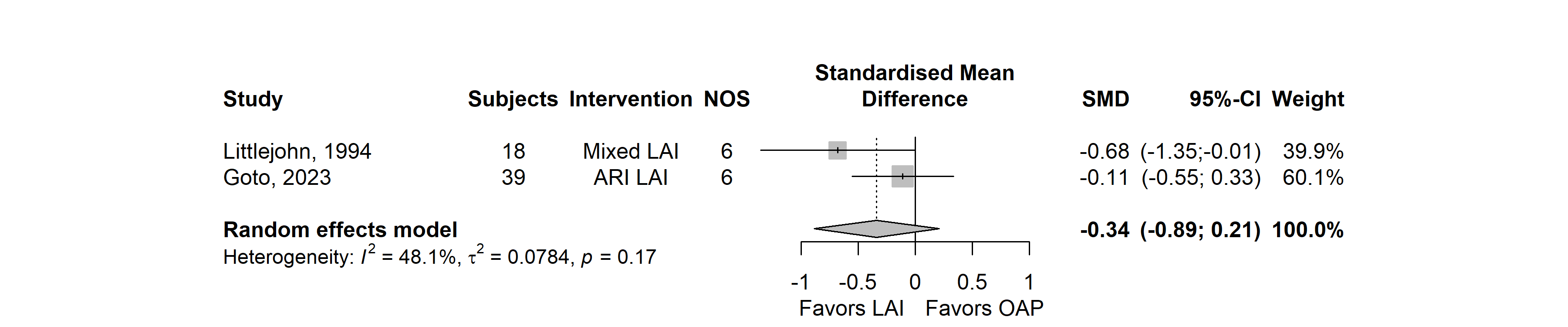
**

Abbreviations: AP = antipsychotic, ARI = aripiprazole, CI = confidence interval, LAI = long-acting injectable, NOS = Newcastle-Ottawa Scale, SMD = standardised mean difference.

**Supplementary Figure 2.** Mean days of hospitalization – mania in mirror-image studies.


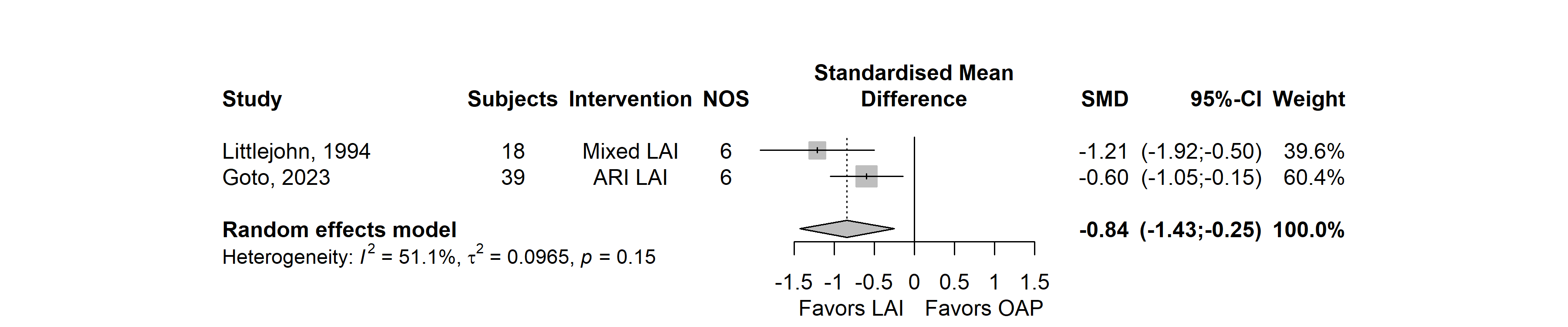


Abbreviations: AP = antipsychotic, ARI = aripiprazole, CI = confidence interval, LAI = long-acting injectable, NOS = Newcastle-Ottawa Scale, SMD = standardised mean difference.

**Supplementary Figure 3.** Mean days of hospitalization – psychiatric in mirror-image studies.


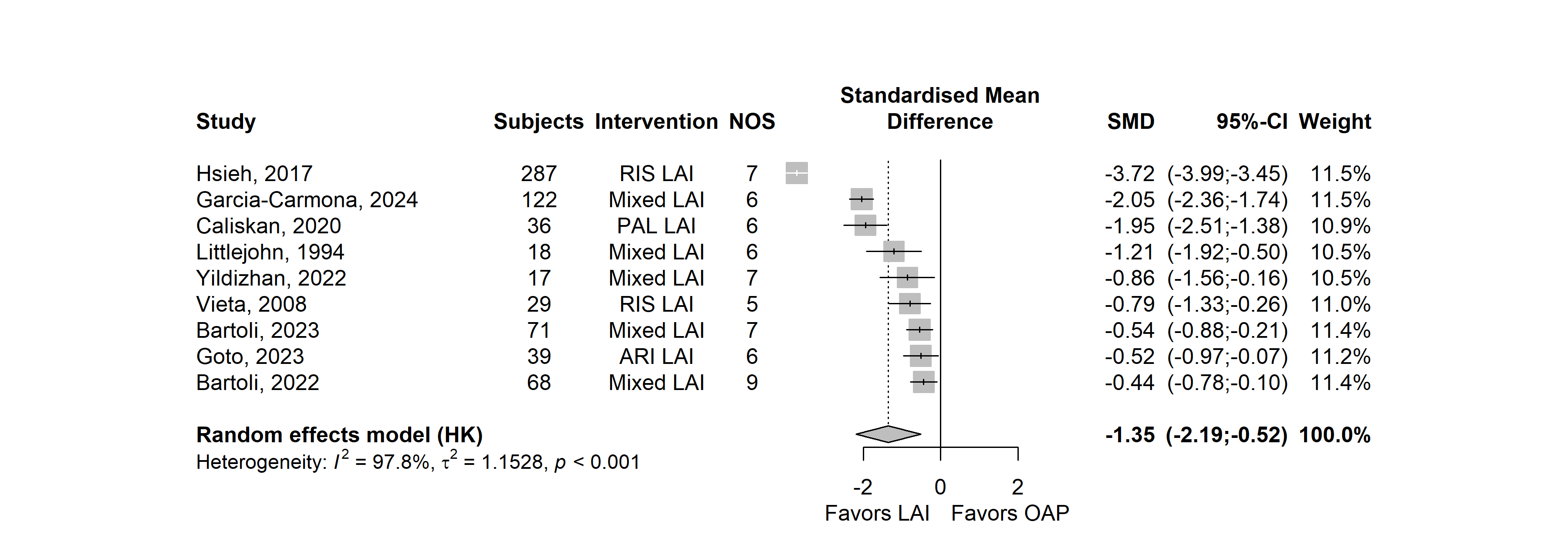


Abbreviations: AP = antipsychotic, ARI = aripiprazole, CI = confidence interval, LAI = long-acting injectable, NOS = Newcastle-Ottawa Scale, PAL = paliperidone, RIS = risperidone, SMD = standardised mean difference.

**Supplementary Figure 4.** Mean number of ED visits in cohort studies.


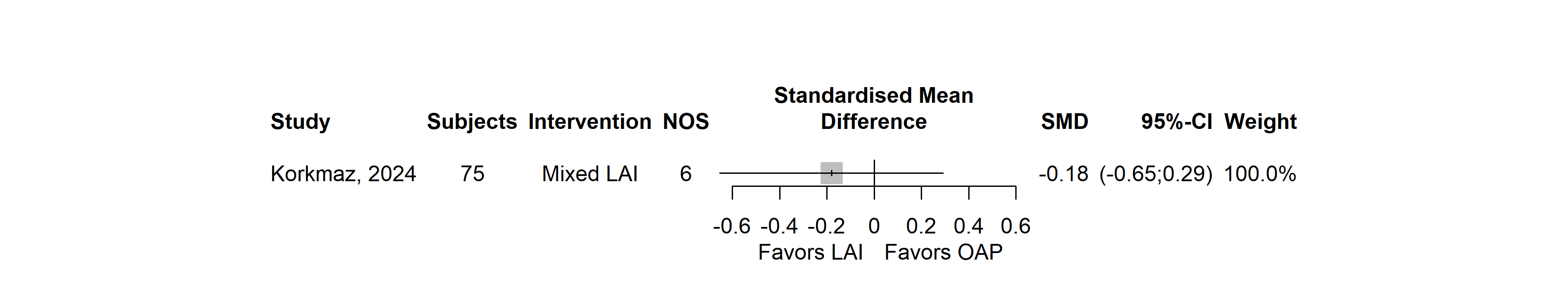
Abbreviations: AP = antipsychotic, CI = confidence interval, LAI = long-acting injectable, NOS = Newcastle-Ottawa Scale, SMD = standardised mean difference.

**Supplementary Figure 5.** Mean number of ED visits in mirror-image studies.


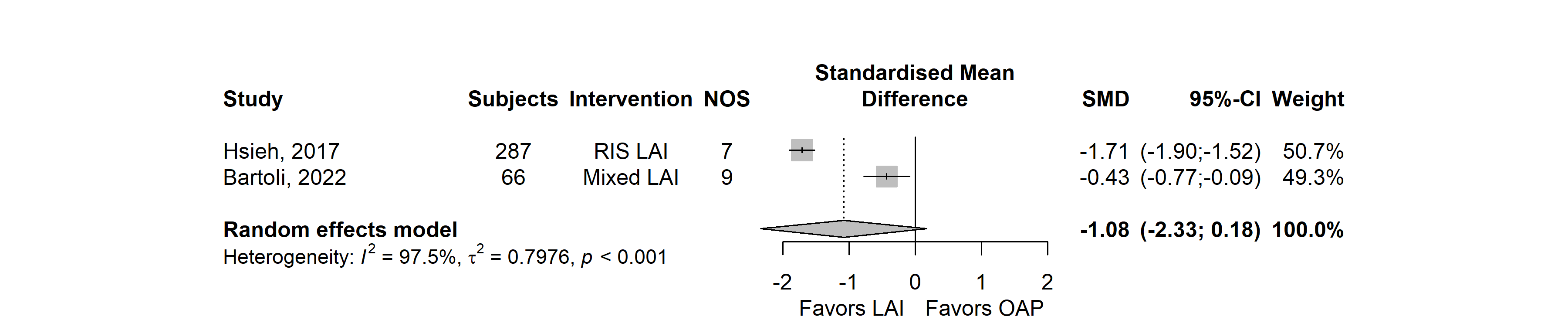


Abbreviations: AP = antipsychotic, CI = confidence interval, LAI = long-acting injectable, NOS = Newcastle-Ottawa Scale, RIS = risperidone, SMD = standardised mean difference.

**Supplementary Figure 6.** Mean number of depressive mood episodes in mirror-image studies.

**
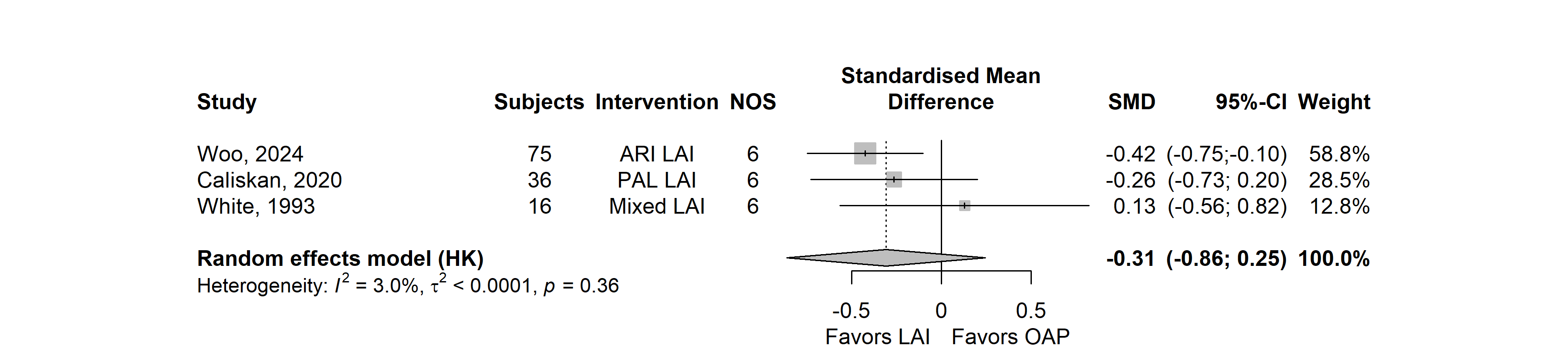
**

Abbreviations: AP = antipsychotic, ARI = aripiprazole, CI = confidence interval, LAI = long-acting injectable, NOS = Newcastle-Ottawa Scale, PAL = paliperidone, SMD = standardised mean difference.

**Supplementary Figure 7.** Mean number of manic mood episodes in mirror-image studies.

**
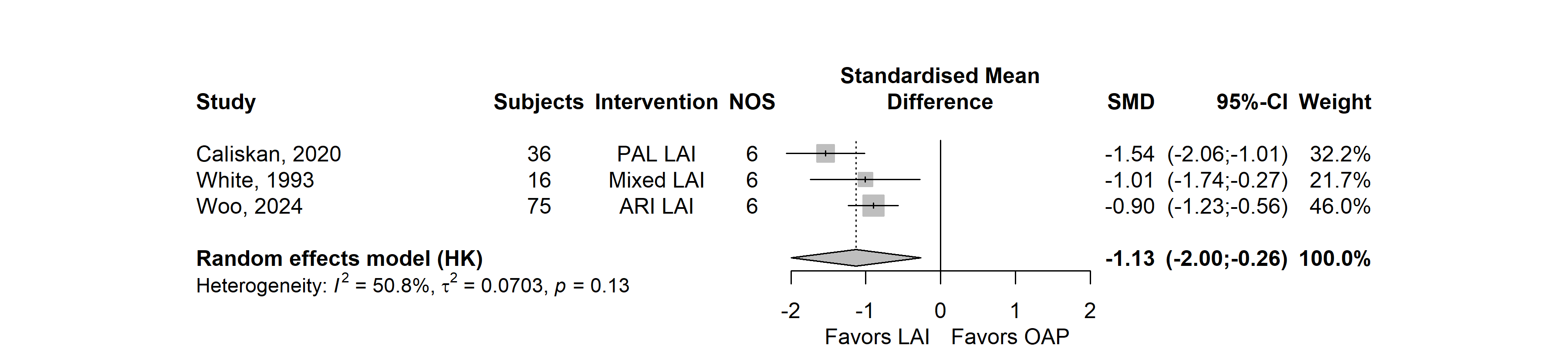
**

Abbreviations: AP = antipsychotic, ARI = aripiprazole, CI = confidence interval, LAI = long-acting injectable, NOS = Newcastle-Ottawa Scale, PAL = paliperidone, SMD = standardised mean difference.

**Supplementary Figure 8.** Mean number of mixed mood episodes in mirror-image studies.


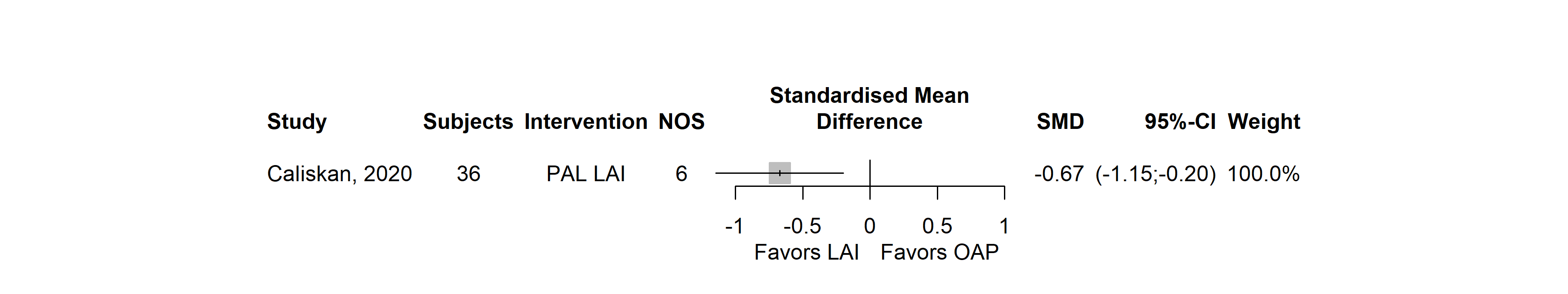


Abbreviations: AP = antipsychotic, CI = confidence interval, LAI = long-acting injectable, NOS = Newcastle-Ottawa Scale, PAL = paliperidone, SMD = standardised mean difference.

**Supplementary Figure 9.** Mean number of any mood episodes in mirror-image studies.

**
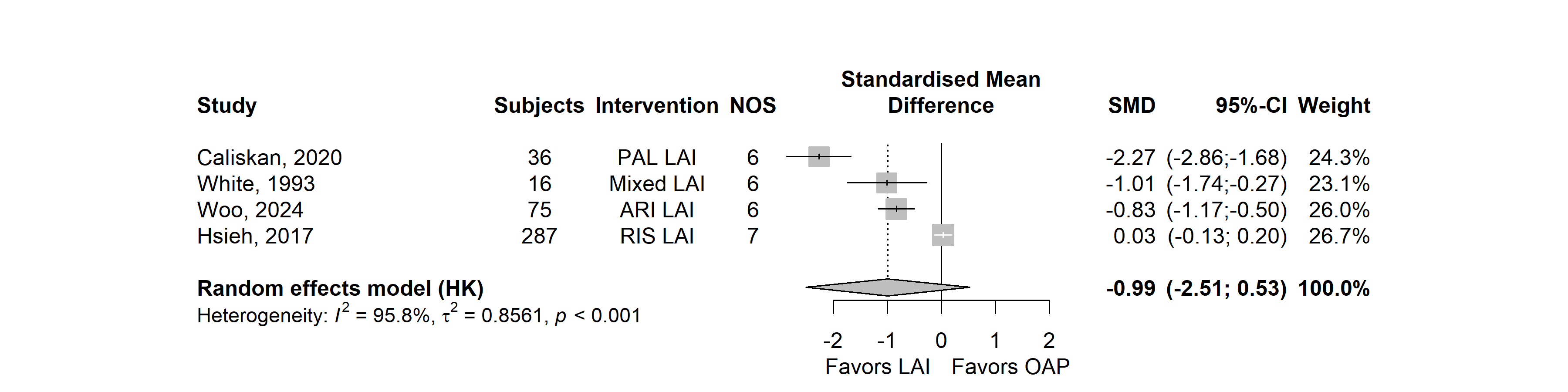
**

Abbreviations: AP = antipsychotic, ARI = aripiprazole, CI = confidence interval, LAI = long-acting injectable, NOS = Newcastle-Ottawa Scale, PAL = paliperidone, RIS = risperidone, SMD = standardised mean difference.

**Supplementary Figure 10.** Mean number of hospitalizations due to depression in mirror-image studies.

**
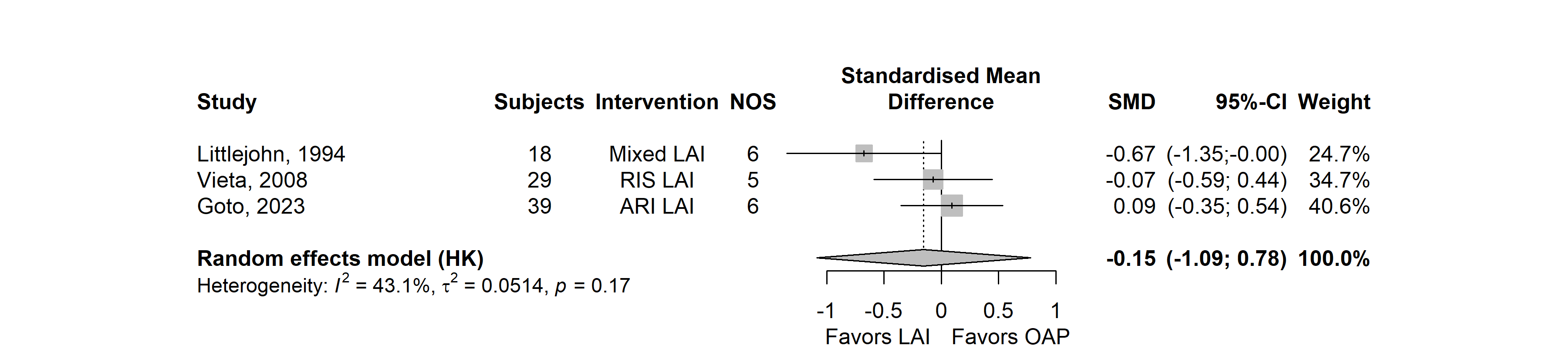
**

Abbreviations: AP = antipsychotic, ARI = aripiprazole, CI = confidence interval, LAI = long-acting injectable, NOS = Newcastle-Ottawa Scale, RIS = risperidone, SMD = standardised mean difference.

**Supplementary Figure 11.** Mean number of hospitalizations due to mania in mirror-image studies.

**
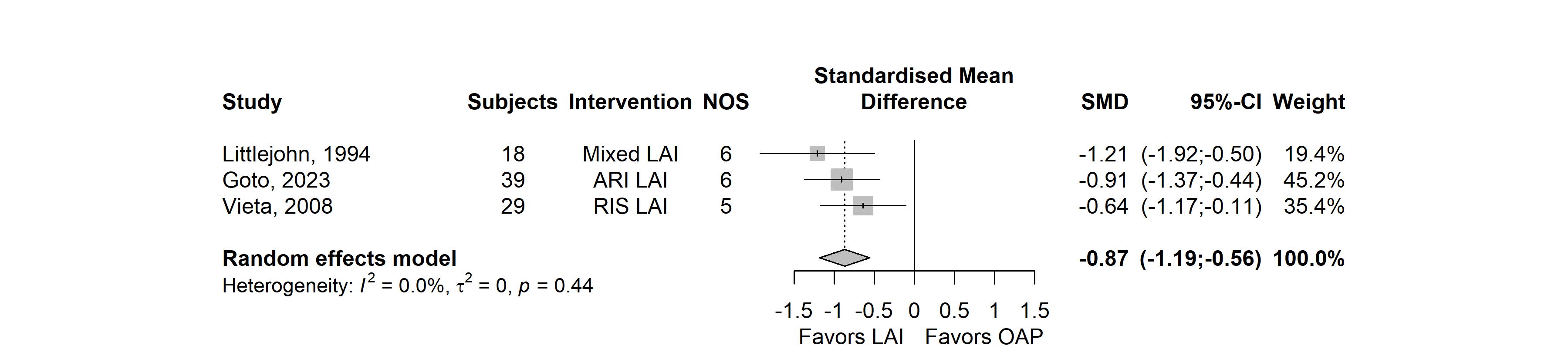
**

Abbreviations: AP = antipsychotic, ARI = aripiprazole, CI = confidence interval, LAI = long-acting injectable, NOS = Newcastle-Ottawa Scale, RIS = risperidone, SMD = standardised mean difference.

**Supplementary Figure 12.** Mean number of psychiatric hospitalizations in cohort studies.

**
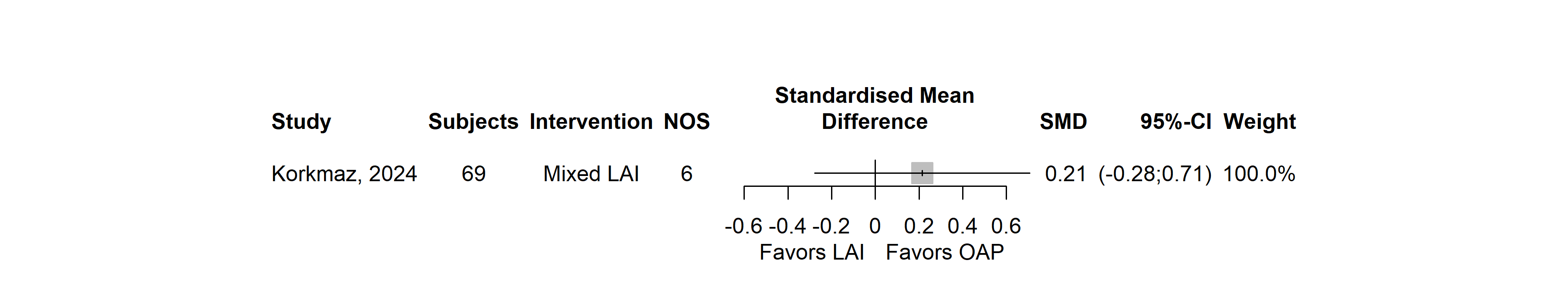
**

Abbreviations: AP = antipsychotic, CI = confidence interval, LAI = long-acting injectable, NOS = Newcastle-Ottawa Scale, SMD = standardised mean difference.

**Supplementary Figure 13.** Mean number of psychiatric hospitalizations in mirror-image studies.


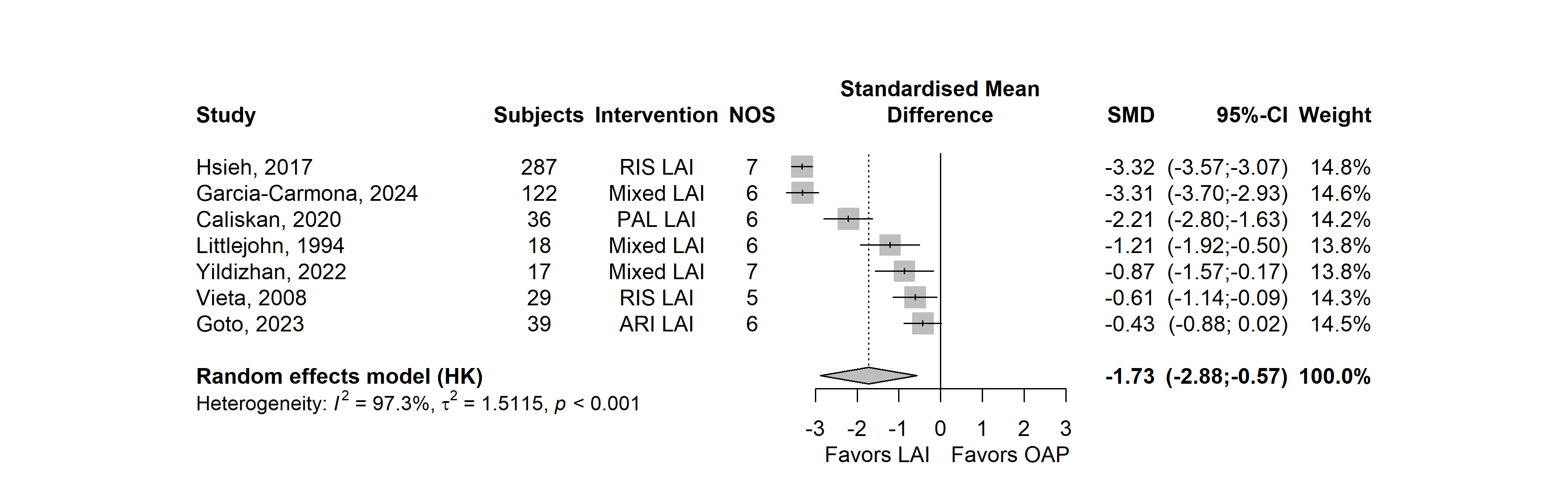


Abbreviations: AP = antipsychotic, ARI = aripiprazole, CI = confidence interval, LAI = long-acting injectable, NOS = Newcastle-Ottawa Scale, PAL = paliperidone, RIS = risperidone, SMD = standardised mean difference.

**Supplementary Figure 14.** Mean time to relapse (days) in mirror-image studies.

**
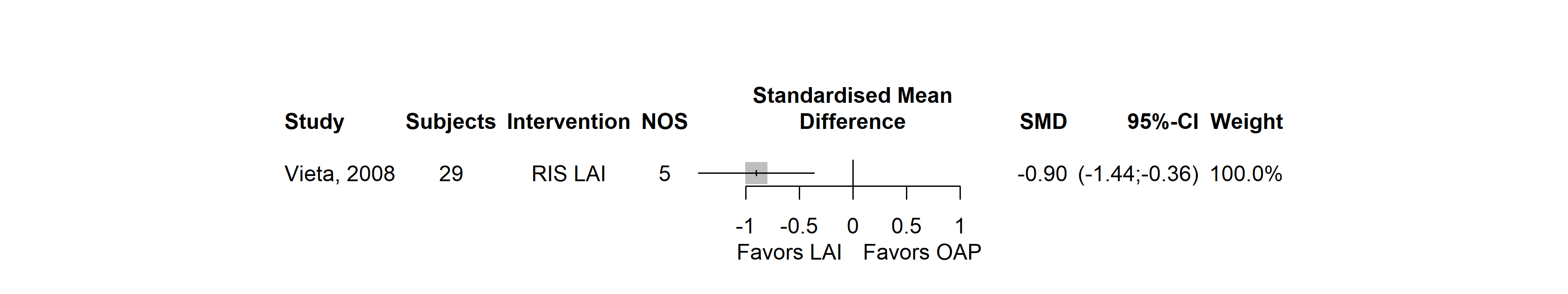
**

Abbreviations: AP = antipsychotic, CI = confidence interval, LAI = long-acting injectable, NOS = Newcastle-Ottawa Scale, RIS = risperidone, SMD = standardised mean difference.

**Supplementary Figure 15.** Risk of hospitalization – psychiatric or non-psychiatric in mirror-image studies.

**
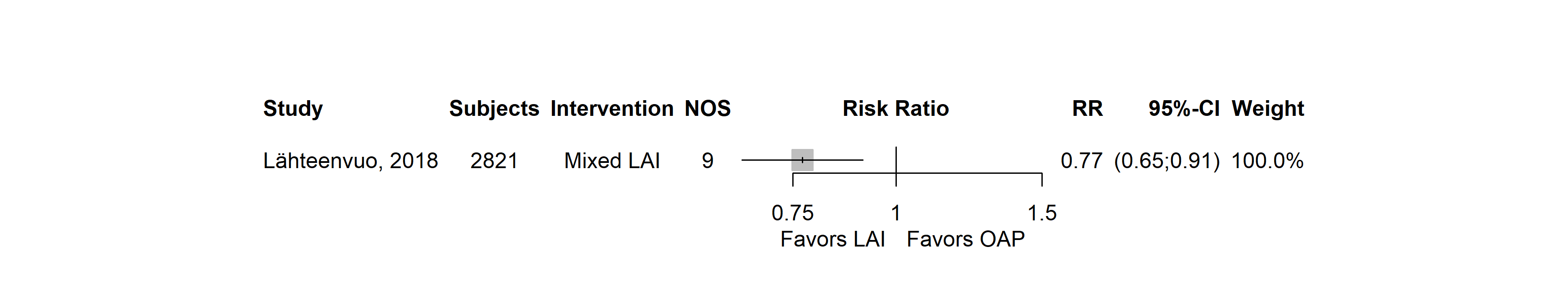
**

Abbreviations: AP = antipsychotic, CI = confidence interval, LAI = long-acting injectable, NOS = Newcastle-Ottawa Scale, RR = risk ratio.

**Supplementary Figure 16.** Risk of emergency department visit in mirror-image studies.

**
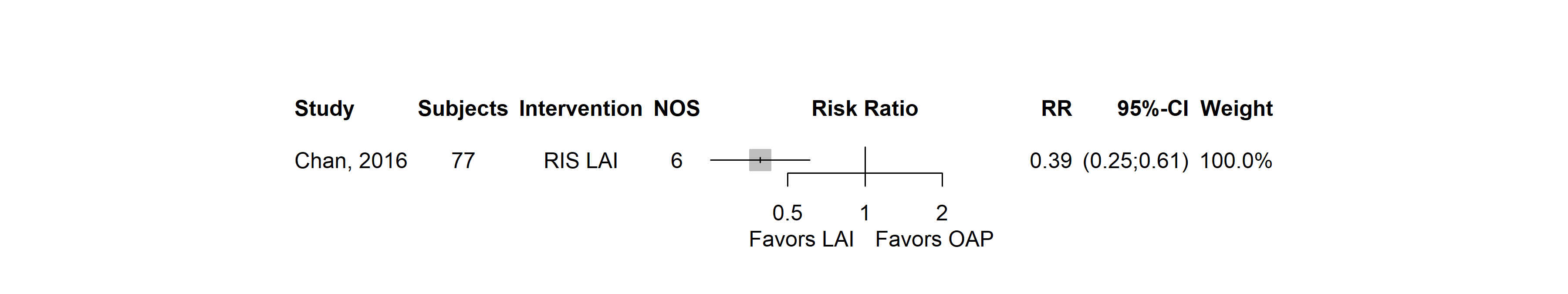
**

Abbreviations: AP = antipsychotic, CI = confidence interval, LAI = long-acting injectable, NOS = Newcastle-Ottawa Scale, RIS = risperidone, RR = risk ratio.

**Supplementary Figure 17.** Risk of hospitalization due to depression in mirror-image studies.


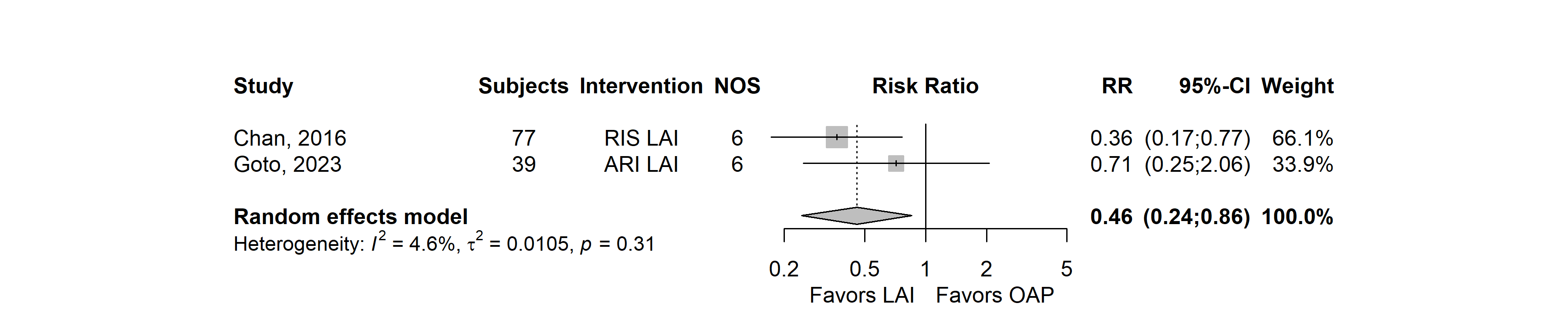


Abbreviations: AP = antipsychotic, ARI = aripiprazole, CI = confidence interval, LAI = long-acting injectable, NOS = Newcastle-Ottawa Scale, RIS = risperidone, RR = risk ratio.

**Supplementary Figure 18.** Risk of psychiatric hospitalization in mirror-image studies.


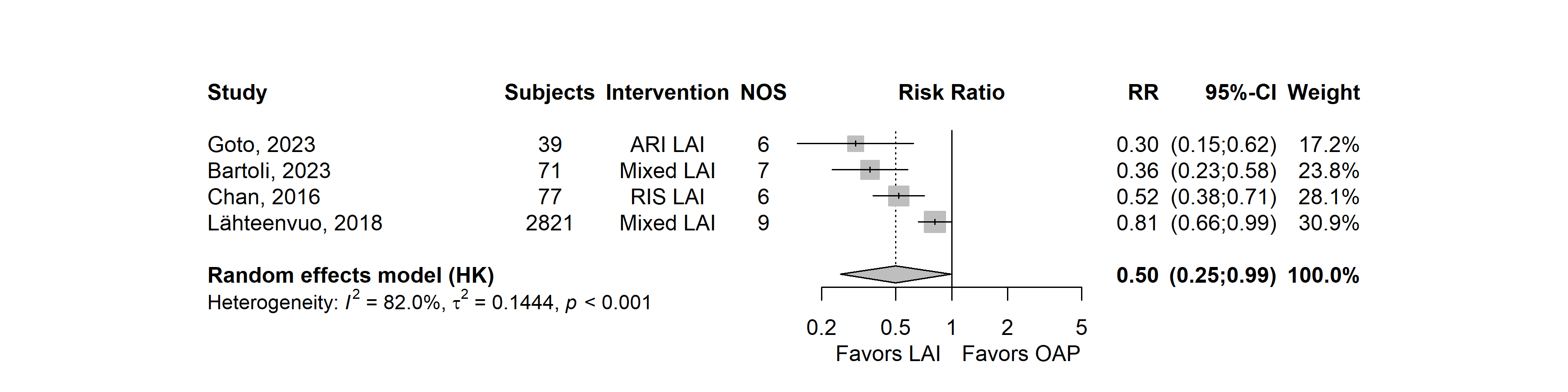


Abbreviations: AP = antipsychotic, ARI = aripiprazole, CI = confidence interval, LAI = long-acting injectable, NOS = Newcastle-Ottawa Scale, RIS = risperidone, RR = risk ratio.

**Supplementary Figure 19.** Risk of psychiatric hospitalization in cohort studies.

**
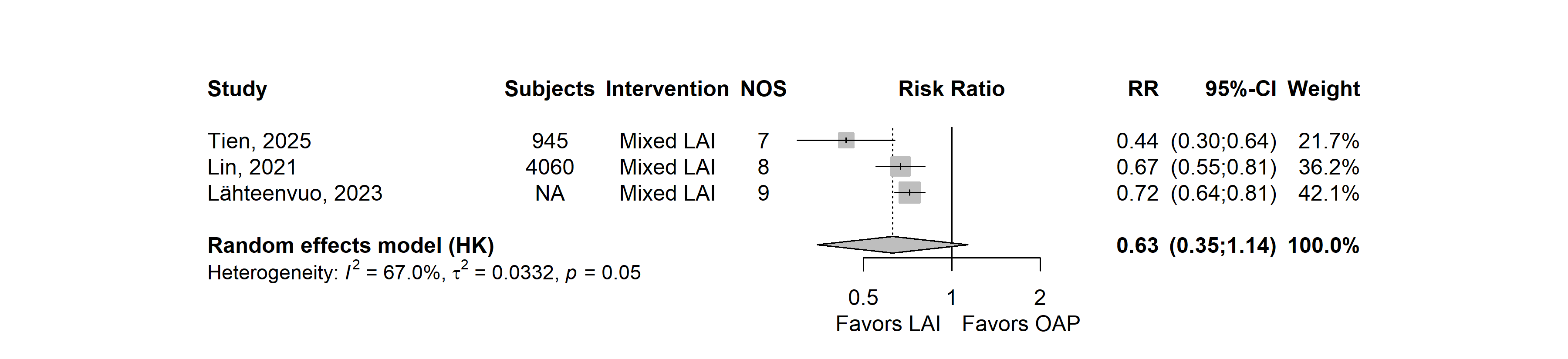
**

Abbreviations: AP = antipsychotic, CI = confidence interval, LAI = long-acting injectable, NOS = Newcastle-Ottawa Scale, RR = risk ratio.

**Supplementary Figure 20.** Risk of non-psychiatric hospitalization in cohort studies.

**
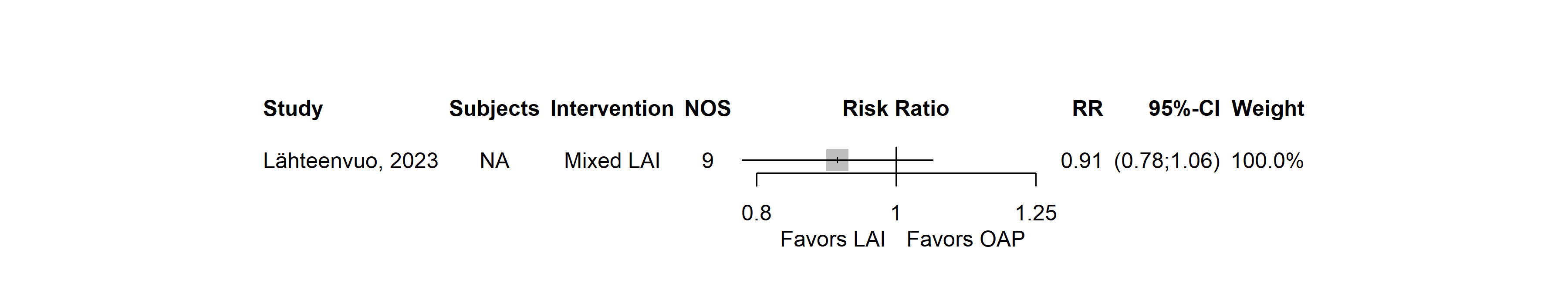
**

Abbreviations: AP = antipsychotic, CI = confidence interval, LAI = long-acting injectable, NOS = Newcastle-Ottawa Scale, RR = risk ratio.

**Supplementary Figure 21.** Risk of hospitalization due to mania in mirror-image studies.

**
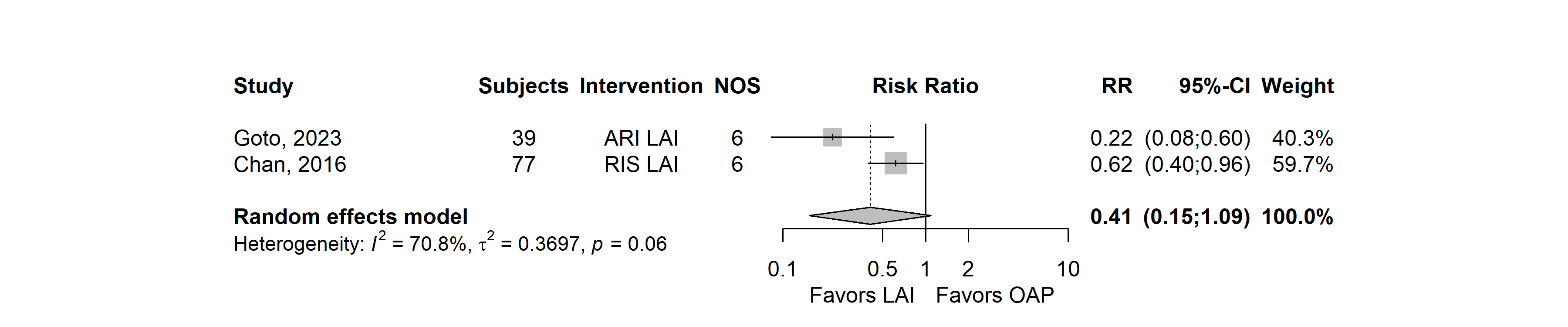
**

Abbreviations: AP = antipsychotic, ARI = aripiprazole, CI = confidence interval, LAI = long-acting injectable, NOS = Newcastle-Ottawa Scale, RIS = risperidone, RR = risk ratio.

**Supplementary Figure 22.** Risk of hospitalization due to mania in cohort studies**.**

**
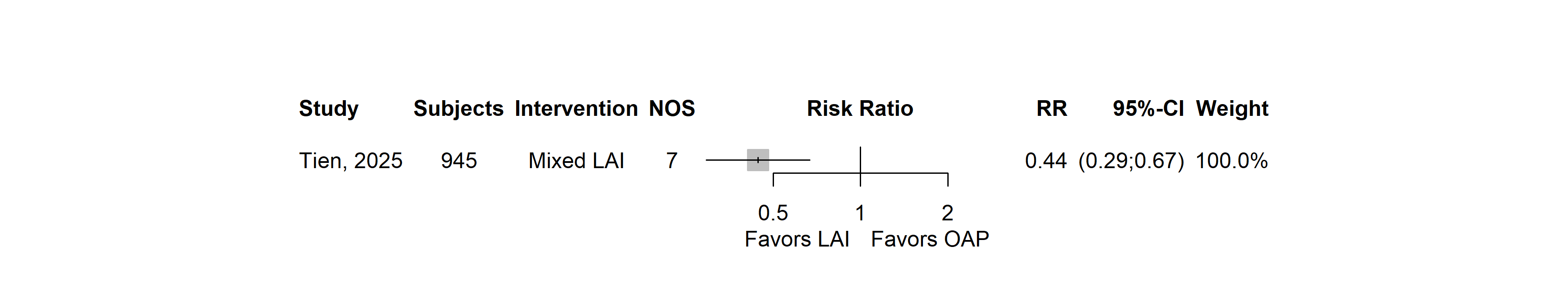
**

Abbreviations: AP = antipsychotic, CI = confidence interval, LAI = long-acting injectable, NOS = Newcastle-Ottawa Scale, RR = risk ratio.

**Supplementary Figure 23.** Risk of hospitalization or relapse in cohort studies.

**
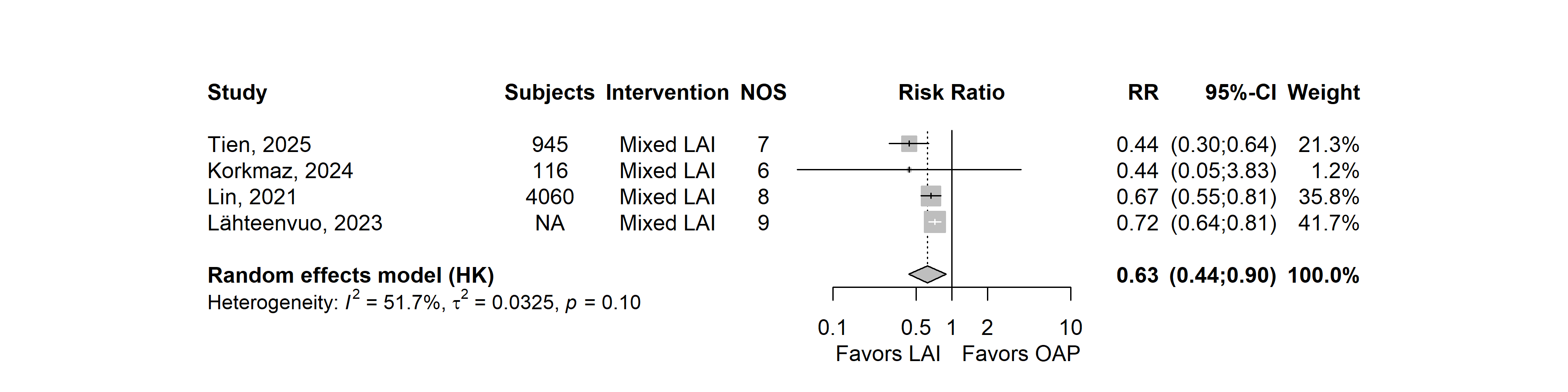
**

Abbreviations: AP = antipsychotic, CI = confidence interval, LAI = long-acting injectable, NOS = Newcastle-Ottawa Scale, RR = risk ratio.

**Supplementary Figure 24.** Risk of hospitalization or relapse in mirror-image studies.

**
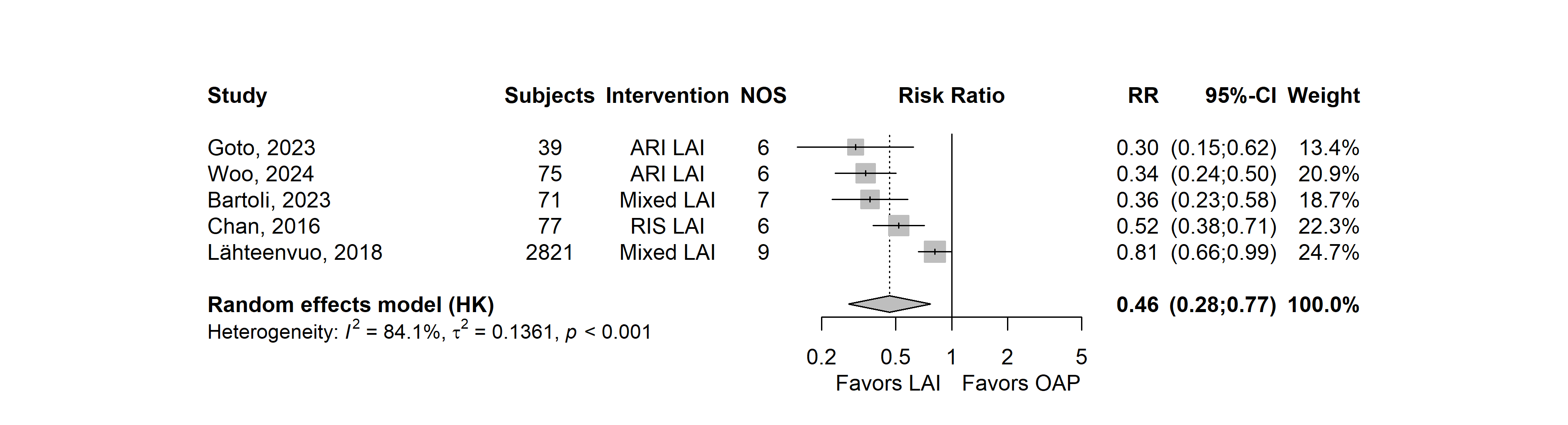
**

Abbreviations: AP = antipsychotic, ARI = aripiprazole, CI = confidence interval, LAI = long-acting injectable, NOS = Newcastle-Ottawa Scale, RIS = risperidone, RR = risk ratio.

**Supplementary Figure 25.** Risk of any relapse in cohort studies.

**
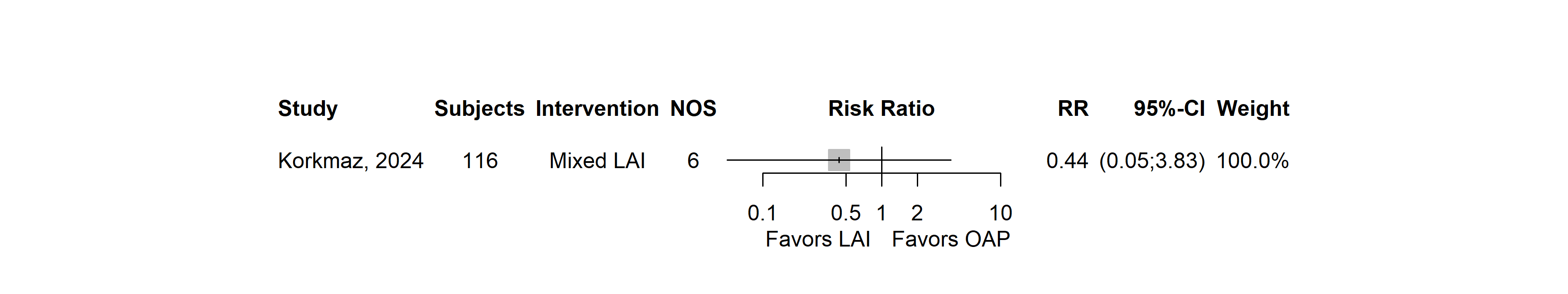
**

Abbreviations: AP = antipsychotic, CI = confidence interval, LAI = long-acting injectable, NOS = Newcastle-Ottawa Scale, RR = risk ratio.

**Supplementary Figure 26.** Risk of any relapse in mirror-image studies.

**
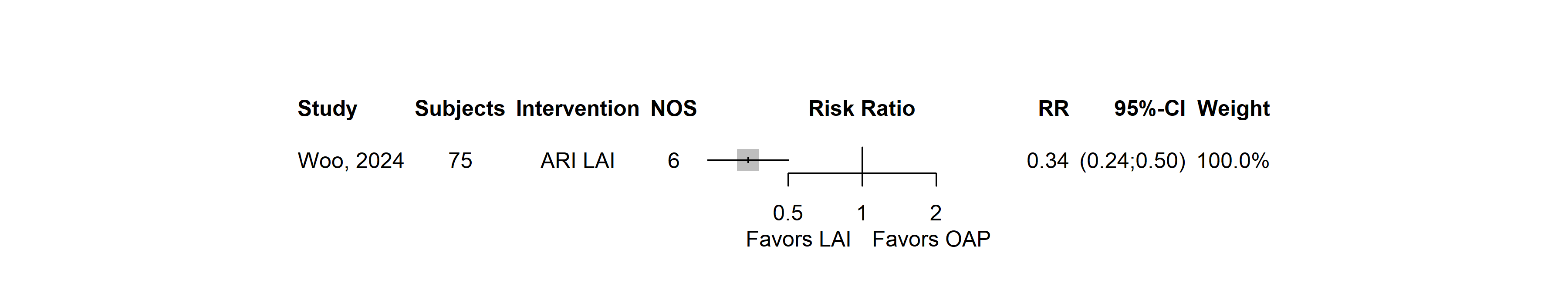
**

Abbreviations: AP = antipsychotic, ARI = aripiprazole, CI = confidence interval, LAI = long-acting injectable, NOS = Newcastle-Ottawa Scale, RR = risk ratio.

**Supplementary Figure 27.** Risk of relapse due to depression in mirror-image studies.

**
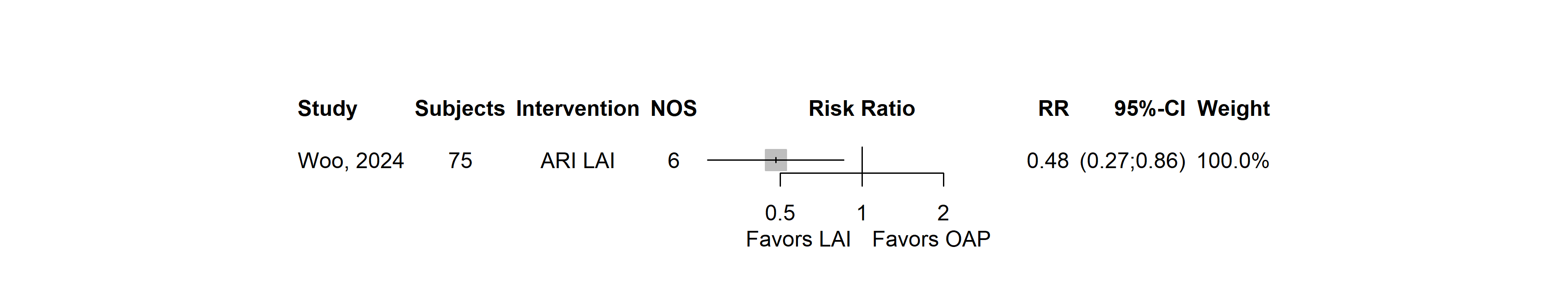
**

Abbreviations: AP = antipsychotic, ARI = aripiprazole, CI = confidence interval, LAI = long-acting injectable, NOS = Newcastle-Ottawa Scale, RR = risk ratio.

**Supplementary Figure 28.** Risk of relapse due to mania in mirror-image studies.

**
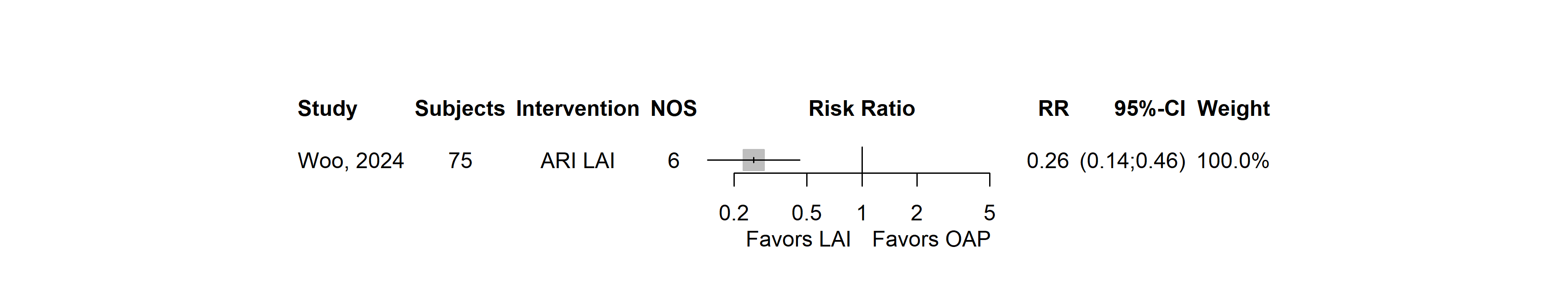
**

Abbreviations: AP = antipsychotic, ARI = aripiprazole, CI = confidence interval, LAI = long-acting injectable, NOS = Newcastle-Ottawa Scale, RR = risk ratio.

**Supplementary Figure 29.** Risk of psychiatric hospitalization by design.

**
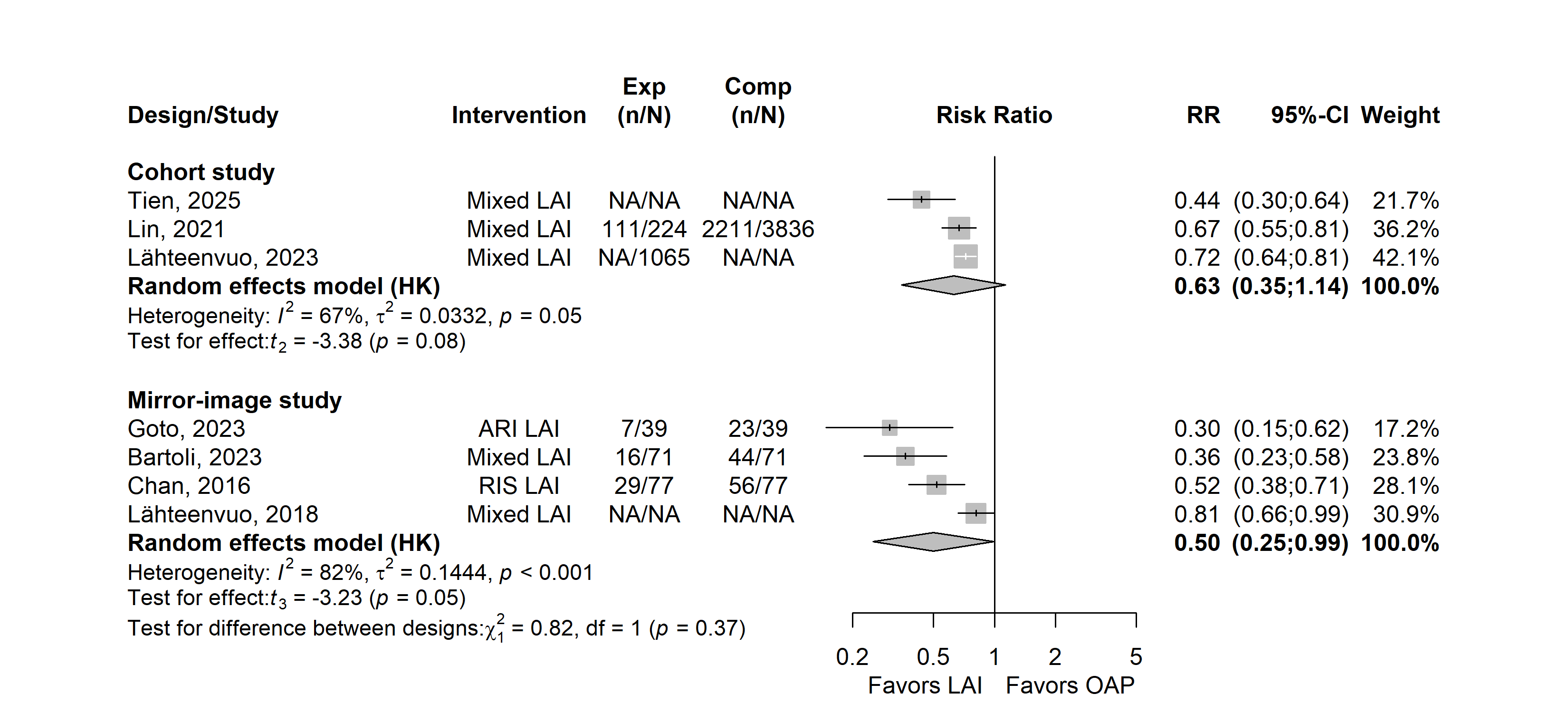
**

Abbreviations: AP = antipsychotic, ARI = aripiprazole, CI = confidence interval, LAI = long-acting injectable, NOS = Newcastle-Ottawa Scale, RIS = risperidone, RR = risk ratio.

**Supplementary Figure 30.** Mean number of episodes by design.

**
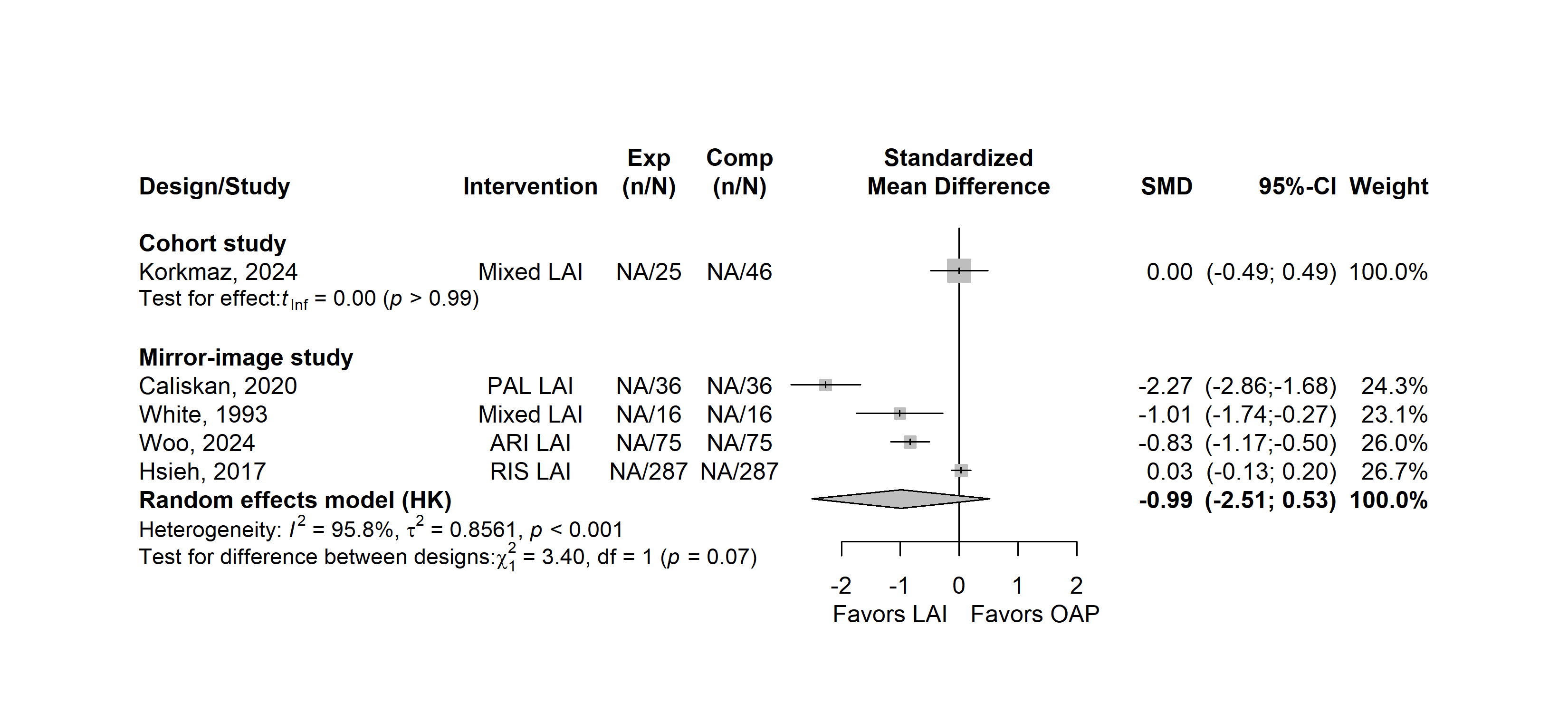
**

Abbreviations: AP = antipsychotic, ARI = aripiprazole, CI = confidence interval, LAI = long-acting injectable, NOS = Newcastle-Ottawa Scale, PAL = paliperidone, RIS = risperidone, SMD = standardized mean difference.

**Supplementary Table 3.** Mean follow-up periods according to respective outcome.

| **Design** | **Outcome** | **k** | **Mean follow-up period in months** | **SD mean follow-up period** |
| --- | --- | --- | --- | --- |
| Cohort study | Mean number of ED visits | 1 | 12 | NA |
| Cohort study | Mean number of episodes - any | 1 | 12 | NA |
| Cohort study | Mean number of hospitalizations - psychiatric | 1 | 12 | NA |
| Cohort study | Risk of hospitalization - non-psychiatric | 1 | 18.1 | NA |
| Cohort study | Risk of hospitalization - mania | 1 | 12 | NA |
| Cohort study | Risk of hospitalization - psychiatric | 3 | 14 | 3.5 |
| Cohort study | Risk of hospitalization or relapse | 4 | 13.5 | 3.1 |
| Cohort study | Risk of relapse - any | 1 | 12 | NA |
| Mirror-image study | Mean days of hospitalization - depression | 2 | 55.2 | 61.1 |
| Mirror-image study | Mean days of hospitalization - mania | 2 | 55.2 | 61.1 |
| Mirror-image study | Mean days of hospitalization - psychiatric | 9 | 24.3 | 28.3 |
| Mirror-image study | Mean number of ED visits | 2 | 12 | 0 |
| Mirror-image study | Mean number of episodes - any | 4 | 23 | 15.1 |
| Mirror-image study | Mean number of episodes - depressive | 3 | 26.6 | 16.1 |
| Mirror-image study | Mean number of episodes - manic | 3 | 26.6 | 16.1 |
| Mirror-image study | Mean number of episodes - mixed | 1 | 12 | NA |
| Mirror-image study | Mean number of hospitalizations - depression | 3 | 44.8 | 46.8 |
| Mirror-image study | Mean number of hospitalizations - mania | 3 | 44.8 | 46.8 |
| Mirror-image study | Mean number of hospitalizations - psychiatric | 7 | 27.8 | 31.7 |
| Mirror-image study | Mean time to relapse (days) | 1 | 24 | NA |
| Mirror-image study | Risk of ED visit | 1 | 12 | NA |
| Mirror-image study | Risk of hospitalization - depression | 2 | 12 | 0 |
| Mirror-image study | Risk of hospitalization - mania | 2 | 12 | 0 |
| Mirror-image study | Risk of hospitalization - psychiatric | 4 | 13.8 | 3.6 |
| Mirror-image study | Risk of hospitalization - psychiatric or non-psychiatric | 1 | 19.2 | NA |
| Mirror-image study | Risk of hospitalization or relapse | 5 | 15.8 | 5.5 |
| Mirror-image study | Risk of relapse - any | 1 | 24 | NA |
| Mirror-image study | Risk of relapse - depression | 1 | 24 | NA |
| Mirror-image study | Risk of relapse - manic | 1 | 24 | NA |

Abbreviations: ED=emergency department, k=number of studies. SD=standard deviation.

**Supplementary Table 4.** Complete statistical results for all outcomes and GRADE assessment.

| **Design/outcome** | **k** | **RR (95% CI)** | **p-value** | **I^2^** | **tau^2^** | **GRADE Quality of Evidence** |
| --- | --- | --- | --- | --- | --- | --- |
| **Cohort studies** |  |  |  |  |  |  |
| Risk of hospitalization - non-psychiatric | 1 | 0.91 (0.78; 1.06) | 0.23 | NA | NA | VERY LOW |
| Risk of hospitalization - mania | 1 | 0.44 (0.29; 0.67) | <0.001 | NA | NA | VERY LOW |
| Risk of hospitalization - psychiatric | 3 | 0.63 (0.35; 1.14) | 0.078 | 67 | 0.03 | VERY LOW |
| Risk of hospitalization or relapse | 4 | 0.63 (0.44; 0.90) | 0.026 | 52 | 0.03 | VERY LOW |
| Risk of relapse - any | 1 | 0.44 (0.05; 3.83) | 0.46 | NA | NA | VERY LOW |
| **Mirror-image studies** |  |  |  |  |  |  |
| Risk of ED visit | 1 | 0.39 (0.25; 0.61) | <0.001 | NA | NA | VERY LOW |
| Risk of hospitalization - depression | 2 | 0.46 (0.24; 0.86) | 0.014 | 5 | 0.01 | LOW |
| Risk of hospitalization - mania | 2 | 0.41 (0.15; 1.09) | 0.075 | 71 | 0.37 | VERY LOW |
| Risk of hospitalization - psychiatric | 4 | 0.50 (0.25; 0.99) | 0.048 | 82 | 0.14 | VERY LOW |
| Risk of hospitalization - any | 1 | 0.77 (0.65; 0.91) | 0.0024 | NA | NA | VERY LOW |
| Risk of hospitalization or relapse | 5 | 0.46 (0.28; 0.77) | 0.013 | 84 | 0.14 | LOW* |
| Risk of relapse - any | 1 | 0.34 (0.24; 0.50) | <0.001 | NA | NA | VERY LOW |
| Risk of relapse - depression | 1 | 0.48 (0.27; 0.86) | 0.013 | NA | NA | VERY LOW |
| Risk of relapse - manic | 1 | 0.26 (0.14; 0.46) | <0.001 | NA | NA | VERY LOW |
| **Design/outcome** | **k** | **MD (95% CI)** | **p-value** | **I^2^** | **tau^2^** |  |
| **Cohort studies** |  |  |  |  |  |  |
| Mean number of ED visits | 1 | -0.13 (-0.43; 0.17) | 0.4 | NA | NA | VERY LOW |
| Mean number of episodes - any | 1 | 0.00 (-0.30; 0.30) | 1 | NA | NA | VERY LOW |
| Mean number of hospitalizations - psychiatric | 1 | 0.12 (-0.18; 0.42) | 0.44 | NA | NA | VERY LOW |
| **Mirror-image studies** |  |  |  |  |  |  |
| Mean days of hospitalization - depression | 2 | -6.59 (-13.20; 0.02) | 0.051 | 0 | 0 | VERY LOW |
| Mean days of hospitalization - mania | 2 | -34.63 (-72.59; 3.32) | 0.074 | 81 | 619.08 | VERY LOW |
| Mean days of hospitalization - psychiatric | 9 | -20.83 (-32.01; -9.65) | 0.0026 | 92 | 122.16 | VERY LOW |
| Mean number of ED visits | 2 | -0.41 (-0.61; -0.20) | <0.001 | 24 | 0.01 | LOW |
| Mean number of episodes - any | 4 | -0.72 (-1.60; 0.17) | 0.081 | 98 | 0.3 | VERY LOW |
| Mean number of episodes - depressive | 3 | -0.09 (-0.48; 0.30) | 0.42 | 64 | 0.01 | VERY LOW |
| Mean number of episodes - manic | 3 | -0.76 (-1.26; -0.25) | 0.023 | 50 | 0.03 | LOW |
| Mean number of episodes - mixed | 1 | -0.19 (-0.32; -0.06) | 0.0046 | NA | NA | VERY LOW |
| Mean number of hospitalizations - depression | 3 | -0.12 (-0.26; 0.02) | 0.085 | 0 | 0 | VERY LOW |
| Mean number of hospitalizations - mania | 3 | -0.66 (-1.25; -0.07) | 0.041 | 20 | 0.02 | LOW |
| Mean number of hospitalizations - psychiatric | 7 | -0.72 (-1.02; -0.41) | 0.0013 | 92 | 0.09 | VERY LOW |
| Mean time to relapse (days) | 1 | -265.40 (-417.03; -113.77) | <0.001 | NA | NA | VERY LOW |
| **Design/outcome** | **k** | **SMD (95% CI)** | **p-value** | **I^2^** | **tau^2^** |  |
| **Cohort studies** |  |  |  |  |  |  |
| Mean number of ED visits | 1 | -0.18 (-0.65; 0.29) | 0.45 | NA | NA | VERY LOW |
| Mean number of episodes - any | 1 | 0.00 (-0.49; 0.49) | 1 | NA | NA | VERY LOW |
| Mean number of hospitalizations - psychiatric | 1 | 0.21 (-0.28; 0.71) | 0.39 | NA | NA | VERY LOW |
| **Mirror-image studies** |  |  |  |  |  |  |
| Mean days of hospitalization - depression | 2 | -0.34 (-0.89; 0.21) | 0.23 | 48 | 0.08 | VERY LOW |
| Mean days of hospitalization - mania | 2 | -0.84 (-1.43; -0.25) | 0.0051 | 51 | 0.1 | LOW |
| Mean days of hospitalization - psychiatric | 9 | -1.35 (-2.19; -0.52) | 0.0058 | 98 | 1.15 | LOW* |
| Mean number of ED visits | 2 | -1.08 (-2.33; 0.18) | 0.092 | 98 | 0.8 | VERY LOW |
| Mean number of episodes - any | 4 | -0.99 (-2.51; 0.53) | 0.13 | 96 | 0.86 | VERY LOW |
| Mean number of episodes - depressive | 3 | -0.31 (-0.86; 0.25) | 0.14 | 3 | 0 | VERY LOW |
| Mean number of episodes - manic | 3 | -1.13 (-2.00; -0.26) | 0.03 | 51 | 0.07 | LOW* |
| Mean number of episodes - mixed | 1 | -0.67 (-1.15; -0.20) | 0.0055 | NA | NA | VERY LOW |
| Mean number of hospitalizations - depression | 3 | -0.15 (-1.09; 0.78) | 0.55 | 43 | 0.05 | VERY LOW |
| Mean number of hospitalizations - mania | 3 | -0.87 (-1.19; -0.56) | <0.001 | 0 | 0 | LOW |
| Mean number of hospitalizations - psychiatric | 7 | -1.73 (-2.88; -0.57) | 0.011 | 97 | 1.51 | LOW* |
| Mean time to relapse (days) | 1 | -0.90 (-1.44; -0.36) | 0.0011 | NA | NA | VERY LOW |

Abbreviations: ED=emergency department, k=number of studies. MD=mean difference, RR=risk ratio, SMD=standardised mean difference.

In GRADE, certainty in evidence from pooled outcomes was downgraded from LOW to VERY LOW in case of high risk of bias (e.g. selection bias), inconsistency (e.g. I^2^>50%) or imprecision (e.g. CIs include both benefit and harm).

*downgraded for inconsistency, upgraded for large and precise estimate – certainty in evidence classified as LOW.

**Supplementary Table 5.** PRISMA 2020 for Abstracts Checklist

**Supplementary Table 6.** PRISMA 2020 Checklist.

**Supplementary Table 7.** Deviations from protocol.

| **Deviation** | **Reason** |
| --- | --- |
| 1. SMD instead of MD for continuous outcomes was used | Strength of association more informative to the reader. MD is reported in supplementary material |
| 1. Hartung-Knapp adjustments for CIs | more robust way to calculate CIs in random-effects meta-analyses, since we could only include few studies |
| 1. Additional covariates for meta-regressions were included | Necessity to explore potential moderators. |
| 1. Primary outcome was changed into hospitalization/relapse instead of hospitalization | Clinically homogenous outcomes |
| 1. Additional secondary outcomes, such as number of mood episodes, hospitalization days were integrated | More comprehensive set of outcomes to improve clinical informativeness |
| 1. Exploratory meta-regressions were not performed | Less than 10 studies were pooled in each analysis |
| 1. Metaconvert was used to transform effect sizes | To use a validated tool for ES conversion |

**Supplementary Table 8.** Sensitivity analyses contrasting the main analysis of mirror-image studies to pre-post correlation coefficients for studies reporting pre and post group means - mean difference (MD) as effect size.

| **Outcome/pre-post correlation** | **N studies** | **MD (95% CI)** | **p-value** | **I²** |
| --- | --- | --- | --- | --- |
| Mean days of hospitalization - depression |  |  |  |  |
| Main analysis | 2 | -6.59 (-13.20; 0.02) | 0.051 | 0 |
| r = 0.3 | 2 | -6.13 (-12.33; 0.06) | 0.052 | 0 |
| r = 0.5 | 2 | -5.68 (-11.43; 0.07) | 0.053 | 0 |
| r = 0.7 | 2 | -5.07 (-10.37; 0.24) | 0.061 | 10 |
| Mean days of hospitalization - mania |  |  |  |  |
| Main analysis | 2 | -34.63 (-72.59; 3.32) | 0.074 | 81 |
| r = 0.3 | 2 | -34.48 (-72.39; 3.44) | 0.075 | 82 |
| r = 0.5 | 2 | -34.37 (-72.26; 3.51) | 0.075 | 82 |
| r = 0.7 | 2 | -34.27 (-72.12; 3.58) | 0.076 | 83 |
| Mean days of hospitalization - psychiatric |  |  |  |  |
| Main analysis | 9 | -20.83 (-32.00; -9.65) | 0.0026 | 92 |
| r = 0.3 | 9 | -21.02 (-32.15; -9.88) | 0.0024 | 93 |
| r = 0.5 | 9 | -21.15 (-32.25; -10.06) | 0.0023 | 94 |
| r = 0.7 | 9 | -21.30 (-32.34; -10.26) | 0.0021 | 96 |
| Mean number of ED visits |  |  |  |  |
| Main analysis | 2 | -0.41 (-0.61; -0.20) | <0.001 | 24 |
| r = 0.3 | 2 | -0.44 (-0.71; -0.18) | <0.001 | 46 |
| r = 0.5 | 2 | -0.47 (-0.76; -0.18) | 0.0015 | 60 |
| r = 0.7 | 2 | -0.49 (-0.79; -0.19) | 0.0016 | 75 |
| Mean number of episodes - any |  |  |  |  |
| Main analysis | 4 | -0.72 (-1.60; 0.17) | 0.081 | 98 |
| r = 0.3 | 4 | -0.72 (-1.60; 0.15) | 0.079 | 98 |
| r = 0.5 | 4 | -0.73 (-1.60; 0.15) | 0.077 | 99 |
| r = 0.7 | 4 | -0.73 (-1.59; 0.14) | 0.075 | 99 |
| Mean number of episodes - depressive |  |  |  |  |
| Main analysis | 3 | -0.09 (-0.48; 0.30) | 0.42 | 64 |
| r = 0.3 | 3 | -0.10 (-0.50; 0.31) | 0.41 | 74 |
| r = 0.5 | 3 | -0.10 (-0.51; 0.31) | 0.4 | 81 |
| r = 0.7 | 3 | -0.10 (-0.52; 0.31) | 0.39 | 88 |
| Mean number of episodes - manic |  |  |  |  |
| Main analysis | 3 | -0.76 (-1.26; -0.25) | 0.023 | 50 |
| r = 0.3 | 3 | -0.76 (-1.26; -0.26) | 0.022 | 65 |
| r = 0.5 | 3 | -0.77 (-1.26; -0.27) | 0.022 | 74 |
| r = 0.7 | 3 | -0.77 (-1.26; -0.28) | 0.021 | 84 |
| Mean number of episodes - mixed |  |  |  |  |
| Main analysis | 1 | -0.19 (-0.32; -0.06) | 0.0046 | NA |
| r = 0.3 | 1 | -0.19 (-0.32; -0.06) | 0.0044 | NA |
| r = 0.5 | 1 | -0.19 (-0.32; -0.06) | 0.0044 | NA |
| r = 0.7 | 1 | -0.19 (-0.32; -0.06) | 0.0044 | NA |
| Mean number of hospitalizations - depression |  |  |  |  |
| Main analysis | 3 | -0.12 (-0.26; 0.02) | 0.085 | 0 |
| r = 0.3 | 3 | -0.11 (-0.38; 0.17) | 0.24 | 0 |
| r = 0.5 | 3 | -0.09 (-0.38; 0.20) | 0.31 | 2 |
| r = 0.7 | 3 | -0.08 (-0.37; 0.22) | 0.38 | 21 |
| Mean number of hospitalizations - mania |  |  |  |  |
| Main analysis | 3 | -0.66 (-1.25; -0.07) | 0.041 | 20 |
| r = 0.3 | 3 | -0.68 (-1.33; -0.04) | 0.045 | 36 |
| r = 0.5 | 3 | -0.71 (-1.40; -0.01) | 0.048 | 47 |
| r = 0.7 | 3 | -0.74 (-1.49; 0.00) | 0.05 | 61 |
| Mean number of hospitalizations - psychiatric |  |  |  |  |
| Main analysis | 7 | -0.72 (-1.02; -0.41) | 0.0013 | 92 |
| r = 0.3 | 7 | -0.72 (-1.04; -0.41) | 0.0013 | 94 |
| r = 0.5 | 7 | -0.73 (-1.05; -0.42) | 0.0012 | 95 |
| r = 0.7 | 7 | -0.74 (-1.06; -0.43) | 0.0012 | 97 |
| Mean time to relapse (days) |  |  |  |  |
| Main analysis | 1 | -265.40 (-417.03; -113.77) | <0.001 | NA |
| r = 0.3 | 1 | -265.40 (-395.66; -135.14) | <0.001 | NA |
| r = 0.5 | 1 | -265.40 (-379.20; -151.60) | <0.001 | NA |
| r = 0.7 | 1 | -265.40 (-359.93; -170.87) | <0.001 | NA |

Abbreviations: ED=emergency department, MD=mean difference.

**Supplementary Table 9.** Sensitivity analyses contrasting the main analysis of mirror-image studies to pre-post correlation coefficients for studies reporting pre and post group means - standardized mean difference (SMD) as effect size.

| **Outcome/pre-post correlation** | **N studies** | **SMD (95% CI)** | **p-value** | **I²** |
| --- | --- | --- | --- | --- |
| Mean days of hospitalization - depression |  |  |  |  |
| Main analysis | 2 | -0.34 (-0.89; 0.21) | 0.23 | 48 |
| r = 0.3 | 2 | -0.31 (-0.87; 0.25) | 0.28 | 59 |
| r = 0.5 | 2 | -0.31 (-0.85; 0.23) | 0.25 | 56 |
| r = 0.7 | 2 | -0.33 (-0.83; 0.18) | 0.21 | 51 |
| Mean days of hospitalization - mania |  |  |  |  |
| Main analysis | 2 | -0.84 (-1.43; -0.25) | 0.0051 | 51 |
| r = 0.3 | 2 | -0.78 (-1.48; -0.08) | 0.029 | 70 |
| r = 0.5 | 2 | -0.81 (-1.43; -0.19) | 0.011 | 62 |
| r = 0.7 | 2 | -0.86 (-1.33; -0.38) | <0.001 | 41 |
| Mean days of hospitalization - psychiatric |  |  |  |  |
| Main analysis | 9 | -1.35 (-2.19; -0.52) | 0.0058 | 98 |
| r = 0.3 | 9 | -1.17 (-1.90; -0.44) | 0.0061 | 98 |
| r = 0.5 | 9 | -1.30 (-2.13; -0.47) | 0.0068 | 98 |
| r = 0.7 | 9 | -1.53 (-2.56; -0.50) | 0.0089 | 98 |
| Mean number of ED visits |  |  |  |  |
| Main analysis | 2 | -1.08 (-2.33; 0.18) | 0.092 | 98 |
| r = 0.3 | 2 | -0.90 (-1.95; 0.16) | 0.095 | 98 |
| r = 0.5 | 2 | -1.05 (-2.29; 0.19) | 0.096 | 98 |
| r = 0.7 | 2 | -1.33 (-2.91; 0.24) | 0.097 | 99 |
| Mean number of episodes - any |  |  |  |  |
| Main analysis | 4 | -0.99 (-2.51; 0.53) | 0.13 | 96 |
| r = 0.3 | 4 | -0.81 (-2.05; 0.43) | 0.13 | 96 |
| r = 0.5 | 4 | -0.95 (-2.41; 0.50) | 0.13 | 96 |
| r = 0.7 | 4 | -1.23 (-3.08; 0.63) | 0.13 | 97 |
| Mean number of episodes - depressive |  |  |  |  |
| Main analysis | 3 | -0.31 (-0.86; 0.25) | 0.14 | 3 |
| r = 0.3 | 3 | -0.23 (-0.72; 0.26) | 0.18 | 28 |
| r = 0.5 | 3 | -0.24 (-0.86; 0.37) | 0.23 | 47 |
| r = 0.7 | 3 | -0.27 (-1.08; 0.54) | 0.29 | 67 |
| Mean number of episodes - manic |  |  |  |  |
| Main analysis | 3 | -1.13 (-2.00; -0.26) | 0.03 | 51 |
| r = 0.3 | 3 | -0.92 (-1.63; -0.20) | 0.032 | 53 |
| r = 0.5 | 3 | -1.07 (-1.94; -0.20) | 0.034 | 64 |
| r = 0.7 | 3 | -1.36 (-2.53; -0.18) | 0.038 | 75 |
| Mean number of episodes - mixed |  |  |  |  |
| Main analysis | 1 | -0.67 (-1.15; -0.20) | 0.0055 | NA |
| r = 0.3 | 1 | -0.46 (-0.81; -0.12) | 0.0081 | NA |
| r = 0.5 | 1 | -0.46 (-0.81; -0.12) | 0.0081 | NA |
| r = 0.7 | 1 | -0.46 (-0.81; -0.12) | 0.0081 | NA |
| Mean number of hospitalizations - depression |  |  |  |  |
| Main analysis | 3 | -0.15 (-1.09; 0.78) | 0.55 | 43 |
| r = 0.3 | 3 | -0.11 (-0.91; 0.70) | 0.63 | 49 |
| r = 0.5 | 3 | -0.11 (-0.94; 0.72) | 0.62 | 50 |
| r = 0.7 | 3 | -0.12 (-0.97; 0.73) | 0.61 | 52 |
| Mean number of hospitalizations - mania |  |  |  |  |
| Main analysis | 3 | -0.87 (-1.19; -0.56) | <0.001 | 0 |
| r = 0.3 | 3 | -0.69 (-1.33; -0.05) | 0.044 | 30 |
| r = 0.5 | 3 | -0.76 (-1.34; -0.18) | 0.03 | 12 |
| r = 0.7 | 3 | -0.86 (-1.12; -0.60) | <0.001 | 0 |
| Mean number of hospitalizations - psychiatric |  |  |  |  |
| Main analysis | 7 | -1.73 (-2.88; -0.57) | 0.011 | 97 |
| r = 0.3 | 7 | -1.53 (-2.61; -0.44) | 0.014 | 98 |
| r = 0.5 | 7 | -1.67 (-2.84; -0.50) | 0.013 | 98 |
| r = 0.7 | 7 | -1.94 (-3.31; -0.57) | 0.014 | 98 |
| Mean time to relapse (days) |  |  |  |  |
| Main analysis | 1 | -0.90 (-1.44; -0.36) | 0.0011 | NA |
| r = 0.3 | 1 | -0.72 (-1.13; -0.31) | <0.001 | NA |
| r = 0.5 | 1 | -0.83 (-1.25; -0.40) | <0.001 | NA |
| r = 0.7 | 1 | -0.99 (-1.44; -0.55) | <0.001 | NA |

Abbreviations: ED=emergency department, SMD=standardized mean difference.
